# Facial feature prioritization by forensic specialists

**DOI:** 10.1371/journal.pone.0359520

**Published:** 2026-10-21

**Authors:** Carina A. Hahn, P. Jonathon Phillips, John D. Grantham, Amy N. Yates

**Affiliations:** 1 Information Technology Laboratory, National Institute of Standards and Technology, Gaithersburg, Maryland, United States of America; 2 Systems Plus, Inc., Rockville, Maryland, United States of America; Liverpool John Moores University, UNITED KINGDOM OF GREAT BRITAIN AND NORTHERN IRELAND

## Abstract

Forensic face specialists are tasked with comparing facial images to determine identity, a process often guided by protocols emphasizing a comprehensive analysis of the full face. However, the strategies specialists employ regarding feature prioritization and accuracy based on specific features remain under-explored. This study investigated the decision-making processes of forensic face specialists using a novel task where participants matched an occluded probe image to one of two target identities by selectively revealing isolated facial features (e.g., ears, eyes, nose). Feature presentation was either self-selected by the specialist or revealed in a predetermined order based on similarity benchmarks. Results indicate that specialists are highly efficient, frequently achieving accurate identifications while viewing only one or two facial features. Ears were strongly preferred by specialists, but they displayed adaptability, altering their selection strategies based on the similarity of the visible features in the target images. The implications of these findings extend to the training and procedural guidelines for face specialists, highlighting the need for a focused approach to developing identification techniques that enhance accuracy in forensic contexts.

## Introduction

Facial examiners and reviewers, or *face specialists*, perform the critical task of comparing facial images, often indicating how strongly the evidence shows the images are either the same person or different people. Research consistently shows that facial examiners, and some groups of facial reviewers, outperform the general population in the task of facial image comparison [1,2], a proficiency attributed to rigorous training and professional experience rather than innate ability alone [3]. While such comparisons are valuable, the present study focuses specifically on the cognitive processes of experts rather than the performance gap between specialists and novices. The evaluations conducted by forensic face specialists serve as investigative leads and expert testimony in legal proceedings, making the reliability of their methodology a matter of significant legal and scientific interest. While their superior performance is documented, the cognitive strategies they employ to achieve it remain less understood.

To support useful strategies, the Facial Identification Scientific Working Group (FISWG), an international body of experts in face research and forensic practice, provides guidelines for conducting a Morphological Analysis. This method entails conducting a thorough description and comparison of features across the entire face [4,5]. It includes lists of facial features along with terms and methods to describe them in great detail. This guidance directly impacts real-world forensic work. For example, the United States Federal Bureau of Investigation (FBI) Criminal Justice Information Services (CJIS) Division provides training to law enforcement agencies that aligns with FISWG’s morphological analysis guidance [6]. Given its practical applications, we aimed to build upon this guidance by investigating whether certain facial features are more reliable predictors of identity than others, a consideration crucial for improving facial identification accuracy.

Evidence suggests facial features differ in how useful they are to determine identity. Research on *diagnostic features* indicates that specific parts of the face (e.g., ears, scars, or distinct moles) are more predictive of identity than others [7]. However, applying this knowledge is complicated by the fact that humans process faces holistically [8], making it difficult to isolate and assess a single facial feature without the biasing influence of its surroundings [9,10]. This leads to a conflict in potential strategies: should an examiner methodically compare every feature as trained, or should they prioritize the most information-rich features?

Prior work suggests the latter may be effective. Studies on the general population indicate that specific features are more influential for recognition than others [11–16]. Taking this idea further, Towler et al. [16] found that instructing participants to focus on diagnostic features increased identification accuracy, although it also raised false-positive rates. Dunn et al. [12] found that while guiding participants to diagnostic features improved performance, participants could not independently identify those features in a post-test. Therefore, while researchers can empirically determine which features are more useful for a given comparison, people may not be able to determine these on their own. To date, the potential for this type of perceptual awareness has been untested in face specialists.

To address this, we moved beyond surveys, which are often unreliable reflections of cognitive processes [17], and designed a task that forces specialists to explicitly prioritize viewing different facial features. We implemented a gradual reveal process rather than presenting features in total isolation to allow participants to leverage combinations of features if necessary to accumulate visual evidence during identification. With this experimental design, we isolated three critical components of expert decision-making:

1. Efficiency: How much visual information does a specialist need to make a decision?
2. Adaptability: Do specialists change their strategy based on the visual similarity of the target faces?
3. Perceptual sensitivity: Do the features specialists prefer to reveal correspond to the features that lead to accurate decisions?

In this study, we focused on nine features that are a subset of those defined by FISWG (cheeks, chin, ears, eyes, eyebrows, forehead, neck, nose, and mouth, see Fig 3). These are also features tested in [7] which provided consistency with prior research. We note that the diagnostic utility of the features is not fixed. While general trends exist, there is substantial variability for any given feature across different comparisons [14]. This large variability means it is unlikely that any specific feature will be the “most useful” across all faces. To determine if specialists can adapt to this variability — rather than rigidly adhering to a static “best feature” list — we purposefully selected image pairs where specific features were rated as either highly dissimilar (predicting high utility) or highly similar (predicting low utility). By emphasizing feature selection, we aim to determine if the high accuracy of specialists is driven by a rigid adherence to a specific feature set or rather a dynamic evaluation of the visual evidence at hand.

## Benchmarking: feature similarity

Before assessing how specialists use features, we first needed to establish the perceived similarity of the facial features of people in the stimulus set. This baseline allowed us to test specialists’ adaptability in the main experiment by assessing whether the similarity of the target images predicted their feature selection strategies. We conducted a benchmarking study to rate the similarity of specific facial features across the identity pairs used in the main experiment.

### Methods

#### Stimuli.

All face images in this study are a subset of the Triad Identity Matching (TIM) Test [18]. The TIM test is a three-alternative forced-choice task. In its original implementation, participants viewed three face images simultaneously. Two images belonged to the same identity; one presented a different identity (see Fig 1). Participants were tasked with identifying the face that did not match the others. Our rationale for selecting triads from the TIM test was that it allows for an assessment of accuracy, independent of response bias (e.g., a conservative tendency to respond “different identity”). Providing a task that eliminates the effect of response bias was critical for our accuracy assessments because specialists tend to respond conservatively [19], which can obscure their true levels of accuracy when differentiating between same and different identities within a two-alternative forced-choice task.

**Fig 1:**
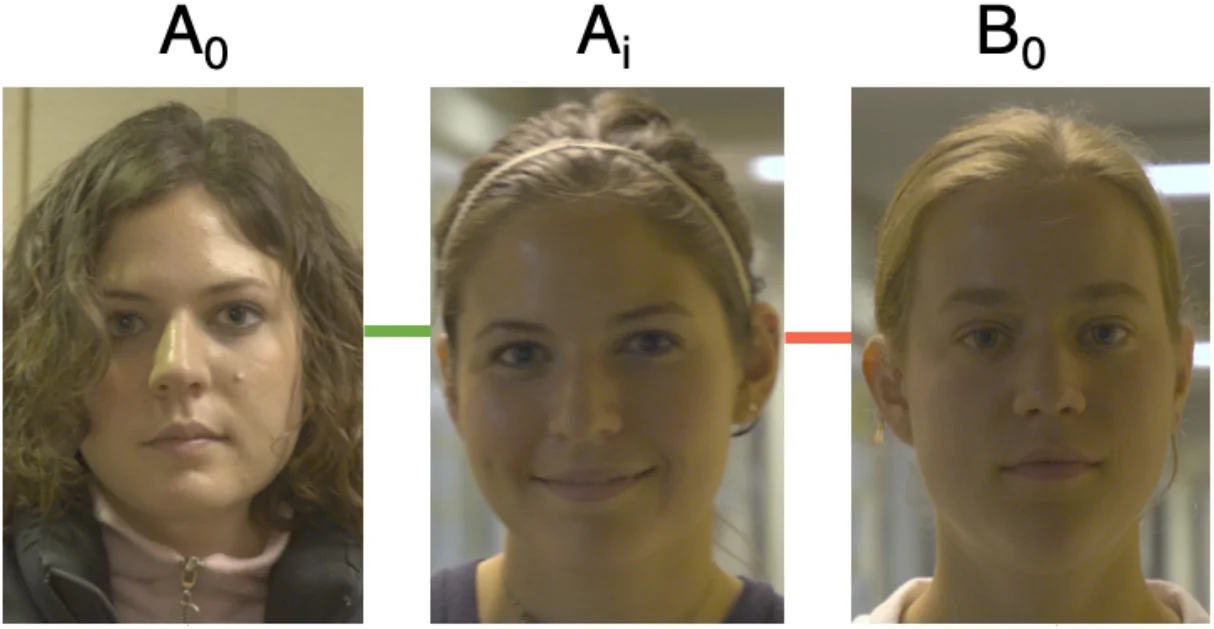
A labeled TIM Test trial. A_0_ and A_i_ are the same identity. *B*_0_ is a different identity. These labels and connector lines are for explanatory purposes only for this figure. Participants only saw the images themselves. The participants’ task was to identify the different identity. In this example, B_0_ is the correct answer. Modified from [18] under a CC BY license, with permission from Behavior Research Methods, original copyright 2023.

Because specialists tend to be more accurate than the general population, we selected images from the TIM test and organized them into target and probe images to make the task challenging for specialists. First, we used the data from [18] to calculate the difficulty of each trial, as measured by calculating the proportion of participants who selected the correct answer. Based on that, we selected the 40 most difficult trials. Our next objective was to ensure that the target images, which always represent different identities, appeared sufficiently similar to be challenging for face specialists. We selected images based on specific difficulty criteria. Because these trials were difficult, the faces representing different identities within the triad often resembled one another more closely than they did the actual matched identity. Consequently, the face within the triad that was most frequently misidentified as the “odd-one-out” (despite actually matching one of the other faces) was designated as the *probe image*. The remaining two images, one matching the probe and one non-matching, served as the *target images*.

We focused on nine facial features: cheeks, chin, ears, eyes, eyebrows, forehead, neck, nose, and mouth. These align with parts of the face described in FISWG guidelines and prior research. The final selection was constrained to features present in most faces (rather than unique markers like moles or scars) that had clear boundaries for segmentation.

#### Procedure.

One of our experimental design objectives was to examine whether inter-identity similarity of the target images predicted feature selections. The target images were always of different people; therefore, these ratings measure coincidental similarity–-the extent to which features appear similar across different identities–-rather than similarity between images of the same person. To obtain these ratings, we recruited non-specialist participants to provide inter-identity similarity ratings of the target images as a benchmark.

In this benchmarking procedure, participants viewed the faces that would be used as target images side-by-side on a computer screen. The faces were fully visible to correspond with the viewing conditions of Experiment 2. For each pair, participants rated the similarity of nine facial features using a 5-point scale: 0 (Not Visible), 1 (Different), 2 (Slightly Different), 3 (Slightly Similar), and 4 (Similar). Participants were instructed to select “Not visible” if a feature was occluded in *either* image. Each participant rated a subset of 20 out of the 40 pairs.

#### Participants.

Participants were recruited from two federal agencies: the National Institute of Standards and Technology (NIST) and the Federal Bureau of Investigation’s Criminal Justice Information Services Division (FBI CJIS). Participants were tested either remotely or in person. Remote ratings were collected using Microsoft Forms, while in-person ratings were gathered using PsychoPy [20]. All participants from NIST participated remotely (n = 10), while those from FBI CJIS were tested in person (n = 24). A total of 34 participants participated over the period from 12 May, 2022–21 Oct, 2022.

For both this benchmarking study and the main experiment, the National Institute of Standards and Technology (NIST) and the Federal Bureau of Investigation (FBI) Institutional Review Boards reviewed and approved the protocol for this project and all subjects, including those depicted in images, provided informed consent in accordance with 15 CFR 27, the Common Rule for the Protection of Human Subjects. Consent was obtained via a button click on the computer screen after participants were provided with a detailed information sheet about the study. People depicted in the images consented to appear in research publications that use the database under a protocol reviewed and approved by the University of Notre Dame. Participants in the images signed a physical consent form prior to participating.

### Results

#### Inter-identity similarity ratings.

We tested whether some features tend to appear more similar across identities than others to establish a benchmark of feature variability. Measuring the coincidental similarity of features between different people allowed us to determine, for specific target identity pairs, which features were more similar and therefore potentially less diagnostic. This enabled us to create predetermined reveal sequences in the main experiment that specifically tested whether specialists adapt their strategies based on the unique diagnostic potential of the features within a given image pair. First, judgments of “not visible” were removed. Next, we measured the average inter-identity similarity rating separately for each facial feature. The pattern of results is shown in Fig 2. Ears were on average rated to be the most perceptually different and necks the most similar. However, there was a large amount of variability for each feature.

**Fig 2:**
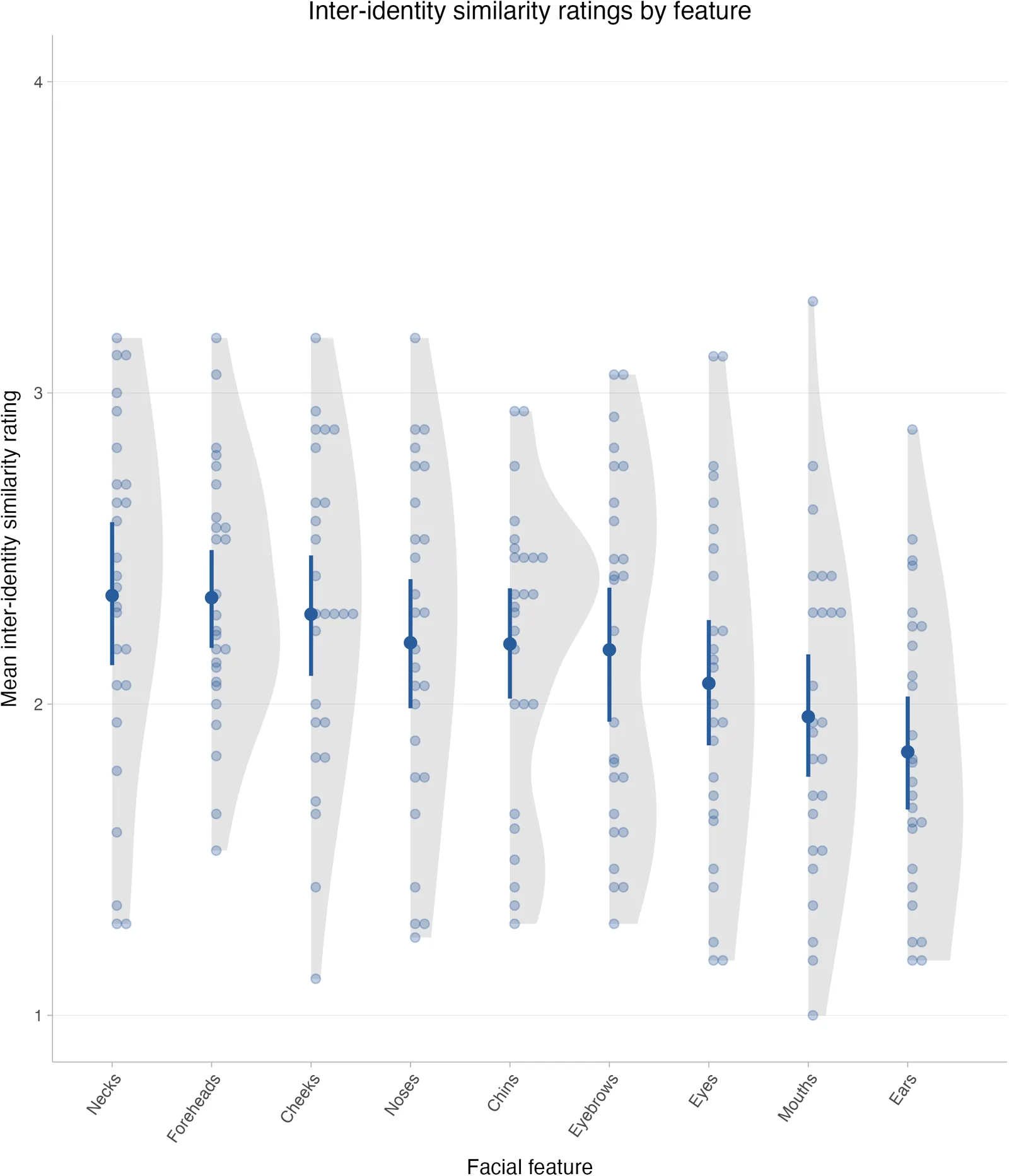
Inter-identity similarity ratings by feature. This plot shows the average inter-identity similarity ratings for each facial feature by image pair after judgments of “not visible” were removed. Individual points represent an image pair. The density of ratings is shown in the shaded half-violin. Large points and error bars show the mean and bootstrapped 95% confidence interval for each feature.

To ensure that inter-identity similarity ratings across participant groups and testing methods were roughly equivalent, we compared the inter-identity similarity judgments of both groups. The results showed that their overall judgments were comparable (Fig A in the Supporting information). As such, we conducted analyses with the two groups combined.

## Experiment: Facial feature use

### Methods

#### Experimental design and procedure.

We designed a study to identify how specialists prioritize the examination of different facial features, and which of these features, when revealed, are associated with improved face matching accuracy. Our design drew inspiration from [21] in that participants revealed parts of an image, in this case a face, before making an identification decision and that a points system incentivized participants to reveal features sparingly. At the start of each trial, participants were presented with two *target images* on a monitor, each depicting a different person. A third image, the *probe image*, displayed a different image of one of the individuals represented in the target images. Participants were tasked with identifying which of the two targets matched the identity of the probe image. Importantly, the probe image was completely obscured when the trial began. We occluded only the probe image and kept the target faces fully visible. To arrive at a decision, participants were allowed to reveal one feature at a time on the probe image until they were ready to make their decision. This design allowed specialists to form a full-face assessment of the target identities first and then selectively reveal features on the probe to verify their assessments about which specific features would be most diagnostic to reveal on the probe image for that particular pair of target images. (Fig 3).

**Fig 3:**
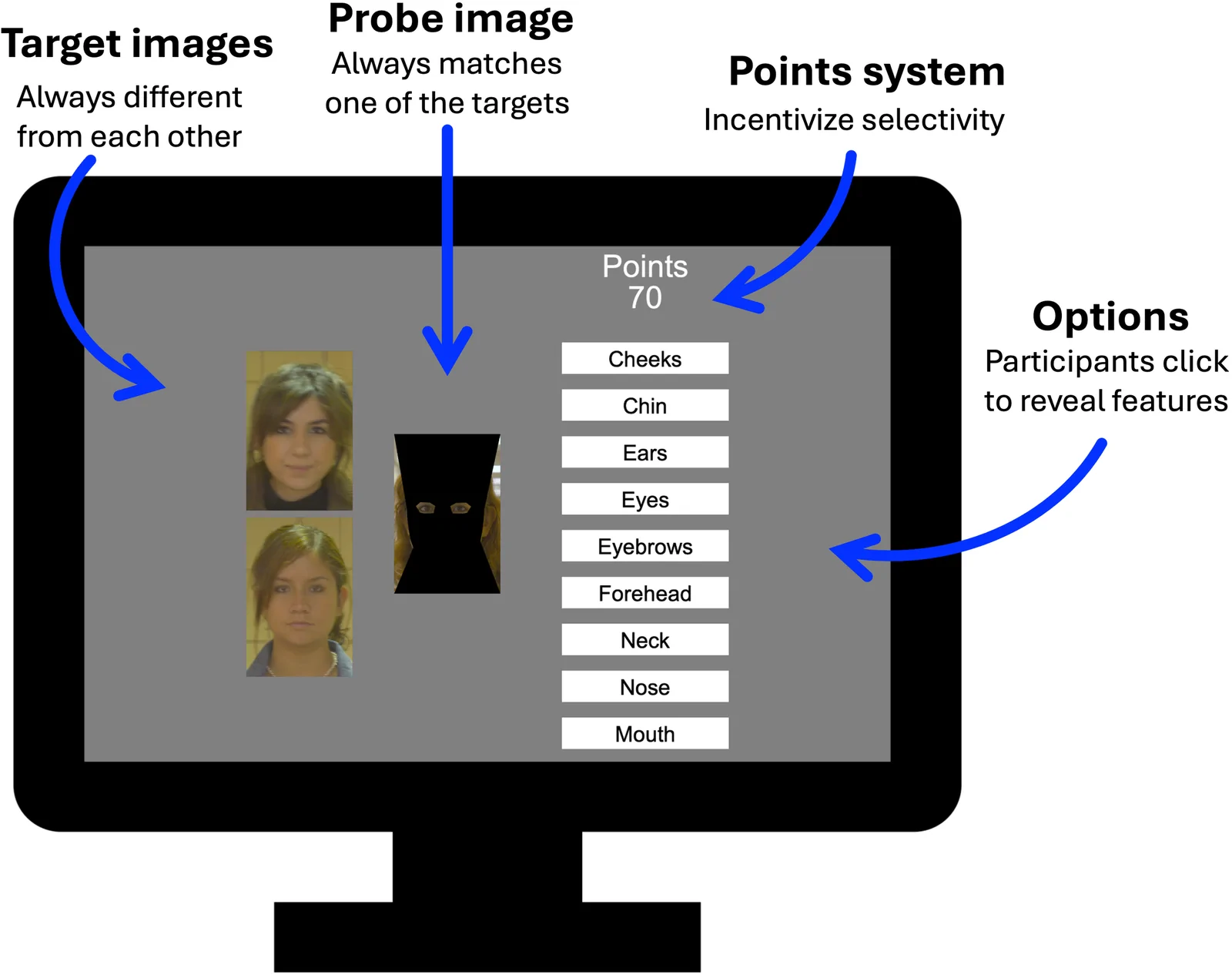
An example experimental trial. An example of an experimental trial in the self-selection condition is shown. On each trial, participants viewed two faces on the computer screen (Target images). They were always of different people. A third face began completely occluded (Probe image). Participants’ task was to match the identity of the probe image to one of the two target images. To make decisions, participants revealed features on the probe (Options). The example shown here corresponds to a participant selecting both Ears and Eyes. A points system was implemented to encourage selective feature reveal decisions and to discourage random guessing. Participants could keep track of their score at the top of the screen (Points system). The predetermined condition was identical except for the available feature options. Participants in that condition had only one button labeled “Reveal.”.

Participants were assigned to one of two between-subjects conditions (*reveal type*). In both conditions, the trial order was randomized for each participant. While the overall procedure was the same for both conditions, the method of revealing features differed. In the *self-selection condition*, participants could choose which features to display on the probe image at their discretion. In the *predetermined condition*, the order in which features were shown was set in advance based on inter-identity similarity ratings from the benchmarking portion of the experiment. In this condition, participants had only one option: “Reveal,” and all other options were removed. Like the self-selection condition, they could decide how many features to reveal, but the sequence was fixed. Within the predetermined condition, we created two within-subjects conditions which differed in the order features were revealed (*sequence type*). In half of the trials, features were revealed starting from the most similar across identities to the most different (*similar first*). In the other half of the trials, the sequence began with the most different feature and ended with the most similar (*dissimilar first*). The sequence type was randomized across trials. In all conditions, participants received one practice trial to get accustomed to the procedure.

These two conditions allowed us to examine multiple aspects of identification decisions. The self-selection condition allowed us to test the preferences of face specialists and whether they changed their preferences based on the similarity of the visible target images. The predetermined condition allowed us to measure the association between the presence of features and accuracy, regardless of preferences.

To ensure accurate interpretations based on the chosen features, we designed a strategy to discourage participants from showing a large number of face features [21]. At the experiment onset, each participant began with a total of 100 points. For every correct response, participants received an additional 100 points. To discourage random guessing, an incorrect answer resulted in a deduction of 200 points. During each trial, participants were permitted to reveal one feature without any loss of points. However, after revealing the initial feature, each subsequent feature selected in that trial resulted in a penalty of 30 points lost. In the instructions, participants were told that their goal was to accumulate as many points as possible.

Therefore, a perfect score with either 0 or 1 feature revealed per trial was 2500: 100+100 points*24 trials. A score based on random guessing with either 0 or 1 feature revealed per trial was −1100: 100+(100 points*12 trials)+(−200 points*12 trials).

At the conclusion of the experiment, participants filled out a brief questionnaire that gathered their sex, age, and other demographic information.

#### Selecting face images.

In the stimuli selection process, we first excluded faces with features that were not visible in the image based on the benchmarking procedure. An image pair was removed from consideration for the final study if it was rated as having any feature “not visible” 50% of the time or more. After excluding those image pairs, 26 remained eligible for the experiment.

After excluding faces with features not visible, we measured the average inter-identity similarity rating for each facial feature within each image pair, excluding judgments of “Not visible.” For each image pair, we ranked the features by their average similarity to determine which were most similar (potentially less diagnostic) and most dissimilar (potentially more diagnostic) for that specific pair. This ranking allowed us to construct the predetermined reveal sequences for the main experiment. To demonstrate that the distribution of similarity was not limited to a small subset of the stimuli, we noted that there were 22 distinct image pairs with at least one feature ranked in the top five most similar, and 18 distinct image pairs with at least one feature ranked in the top five most dissimilar. Because these rankings were feature-specific, the same image pair could be categorized as “similar” for one feature (e.g., eyes) and “different” for another (e.g., mouths).

#### Feature isolation.

To enable the independent presentation of features, we delineated the boundaries of the nine facial regions on each probe image. We utilized the dlib machine learning library [22], pre-trained on the iBUG 300-W dataset [23], to estimate the coordinates of 68 facial landmarks. These coordinates served as the basis for defining the specific boundaries of each feature.

The proposed feature boundaries were presented to FBI project managers and an FBI facial examiner for feedback. The facial examiner was excluded from participating in the study itself due to their involvement in these discussions. We engaged in multiple iterations of refinement based on their assessments until all parties reached a consensus on the appropriateness of the boundaries, leading to their finalization (Fig 4). The focus was on the facial feature regions, and we did not obtain feedback on whether facial details, such as creases, marks, or scars, traversed those boundaries from one facial feature region to another. We note that we sacrificed some precision in the feature boundaries for the sake of having consistent definitions of the coordinates across participants. Because participants would be unable to zoom into images to view minutae or the small details of the features, we felt this trade-off was appropriate in this study. See Section D in the Supporting information for details on how we defined the boundaries of the features.

**Fig 4:**
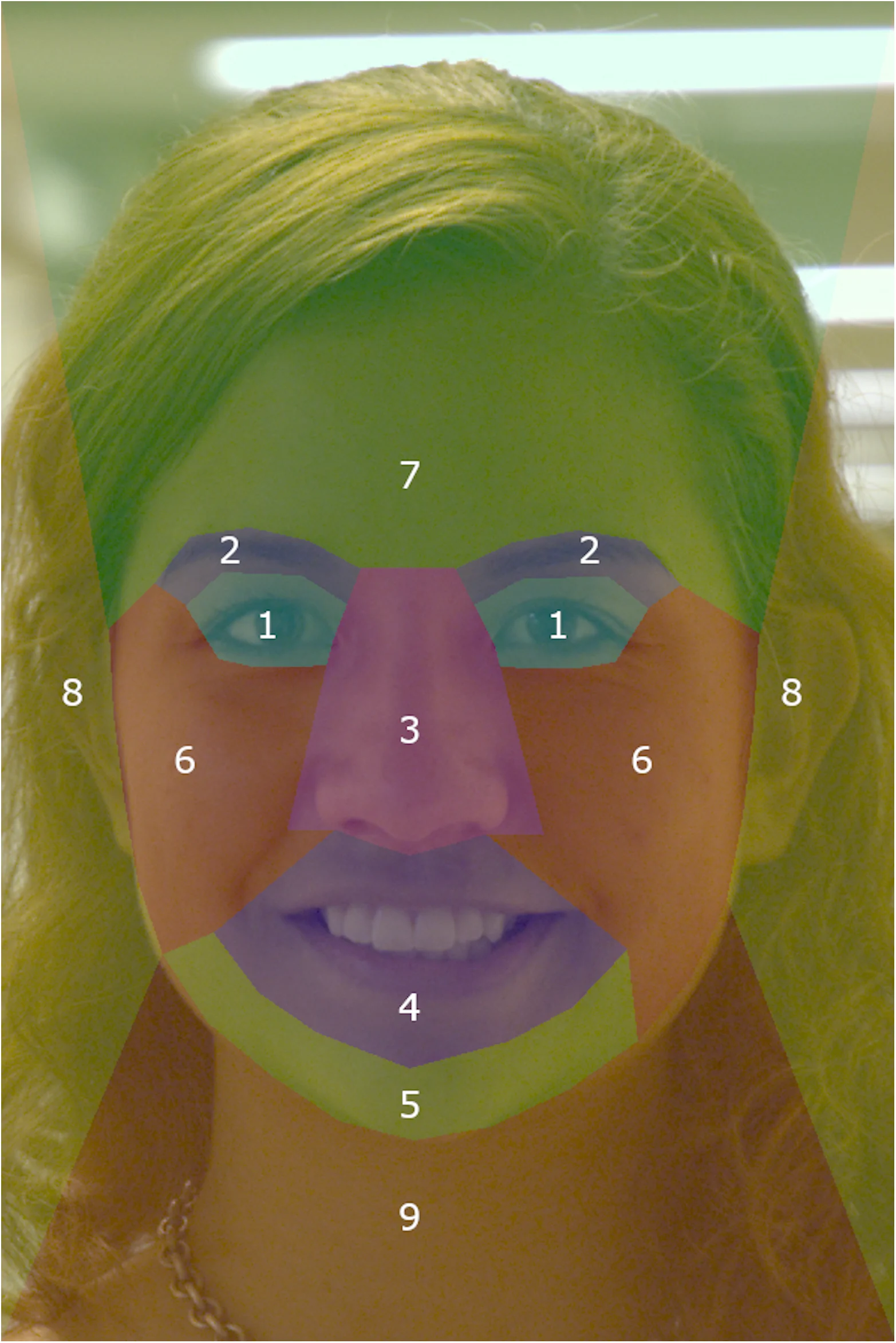
Facial feature boundaries. A face image with labeled shaded regions depicting the areas of the nine facial features defined in this study: 1. Eyes, 2. Eyebrows, 3. Nose, 4. Mouth, 5. Chin, 6. Cheeks, 7. Forehead, 8. Ears, 9. Neck.

#### Participants.

We recruited 27 participants who self-reported that they have face specialist experience and/or training (21 were female; min and max age ranges = 18–29; 60–69). All participants were recruited from federal agencies who employ face specialists between 17 Oct, 2023 and 8 Jul, 2024. Participants were recruited from roles involving facial image comparison, we did not collect data on their specific professional roles (e.g., facial examiner versus reviewer) or their total years of experience.

### Results

#### Accuracy.

Our measure of accuracy is the probability of success (i.e., the correct answer) based on an exact binomial test. This method was selected because the number of trials associated with each factor (both the condition and the number of trials for a given scenario) could vary largely, depending on the analysis presented. For binomial tests for accuracy, we report the probability of success and 95% confidence intervals. First, we assessed potential speed-accuracy trade-offs by calculating the Pearson correlation between success probability and median reaction time for each condition. We found no evidence of a trade-off in any condition (Self-selection: *r*(12) = 0.17, *p* = 0.56; Ordered, dissimilar first: *r*(11) = 0.25, *p* = 0.42; Ordered, similar first: *r*(11) = 0.51, *p* = 0.08).

We focused on accuracy rather than total accumulated points. Because points and accuracy were highly correlated (*r*(38) = 0.5, *p* = 0.001) and the points system served primarily as a behavioral nudge to encourage selectivity, analyzing points provided no additional interpretive insight.

Fig 5 shows the probability of a correct answer for each condition, based on how many features were revealed. We cut off this analysis at four reveals because only 12 trials were made using five or more revealed features. This outcome was expected. By implementing a points system and penalizing reveals beyond the first selection, we aimed to limit the number of features exposed, encouraging more careful and thoughtful decision-making among participants.

**Fig 5:**
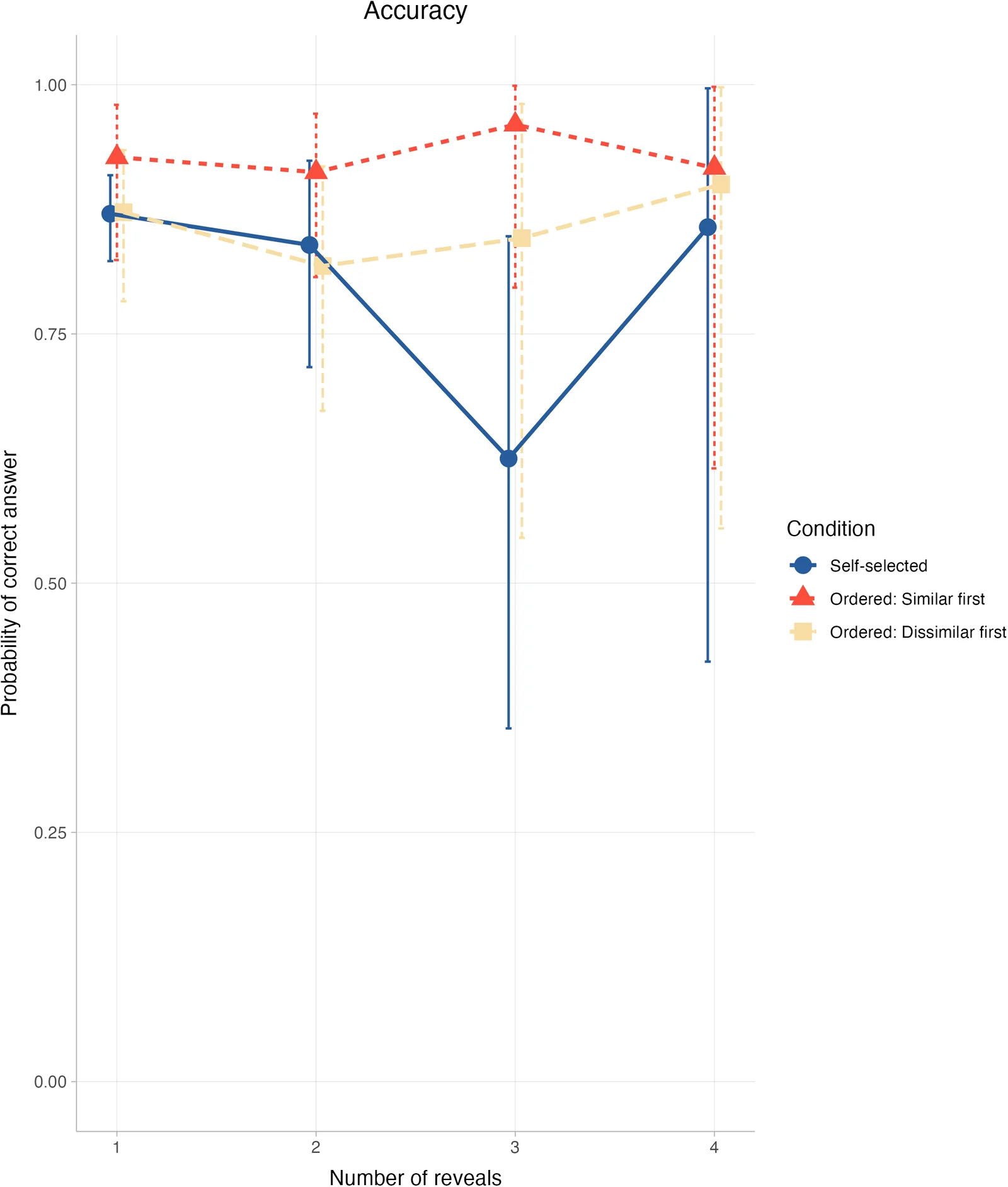
Accuracy by experimental condition and number of features revealed. This graph shows the probability of a correct answer (y-axis) according to exact binomial tests. Points reflect the probability of success. Error bars show the 95% confidence intervals. Accuracy is broken down by the number of features revealed on a trial (x-axis) and experimental condition: self-selected (blue dots, solid line), predetermined ordered by similar features first (red triangles, short dashed line), and predetermined ordered by dissimilar features first (beige squares, long dashed line).

Within each condition, accuracy was largely level across number of reveals. Using a Generalized Linear Mixed-Effects Model (GLMM) with a binomial link function, we found no statistically significant effect of reveal count on success probability in either the Ordered: Dissimilar First or Ordered: Similar First conditions (all Tukey-adjusted p > 0.68). Across these conditions, estimated marginal mean success rates were consistently high, ranging from 91.8% to 98.3%. In the Self-selected condition, the model initially identified a significant difference between the 3-reveal count and the 1-reveal baseline (raw p = 0.0296). However, the Tukey-adjusted pairwise comparisons did not reach the threshold for statistical significance (α=0.05) for any contrast. The most pronounced, though non-significant, effect was observed at the 3-reveal count, where the predicted success probability dropped substantially to 76.9% (compared to ∼95% for other counts). This represented a large effect size, with the odds of success at 1 reveal being 5.3 times higher than at 3 reveals ($p_{adj}$=0.13). Thus, while a strong performance drop was observed at the 3-reveal count in the Self-selected condition, the difference was not statistically reliable after accounting for multiple comparisons.

To achieve steady levels of accuracy, participants revealed different numbers of features at varying rates depending on the experimental condition (Table 1). In the self-selection condition, 75.89% of decisions were made after revealing only one feature, while 16.67% were made after revealing two features. Therefore, when people chose which features to view, they made decisions with only one feature over three-quarters of the time. In contrast, when the feature order was predetermined, the pattern changed. When the most different feature was shown first, participants made decisions after one reveal 55.13% of the time, and 28.21% of the time with two reveals. When similar features were shown first, the pattern changed again: participants made decisions with one feature nearly as often as they made decisions with two features (35.26% and 36.54% respectively).

**Table 1:** Percentage of decisions by features revealed.

Condition	No. of features revealed			
	1	2	3	4
Ordered: Dissimilar first	55%	28%	8%	6%
Ordered: Similar first	35%	37%	16%	8%
Self-selected	76%	17%	5%	2%

Percentage of decisions made after revealing different numbers of features, grouped by experimental condition.

#### Best features for accuracy.

Next, we evaluated which features, when revealed, led to accurate identification decisions. Trials where only a single feature was revealed were the most frequent occurrence, comprising 396 out of a total of 648 trials. Consequently, this analysis is solely on trials with one revealed feature. For details on the selection of feature combinations, see Figs C and D and Section C in the Supporting information.

We calculated the probability of providing a correct answer based on which feature was revealed (Fig 6 as well as Table S1 in S1 File in the Supporting information). In this analysis, we combined both self-selected and predetermined conditions. While there were minor variations depending on whether the feature was shown or used in the self-selected or predetermined conditions, these differences were not substantial, so we will not discuss them further here. For more details, see Fig B in the Supporting information.

**Fig 6:**
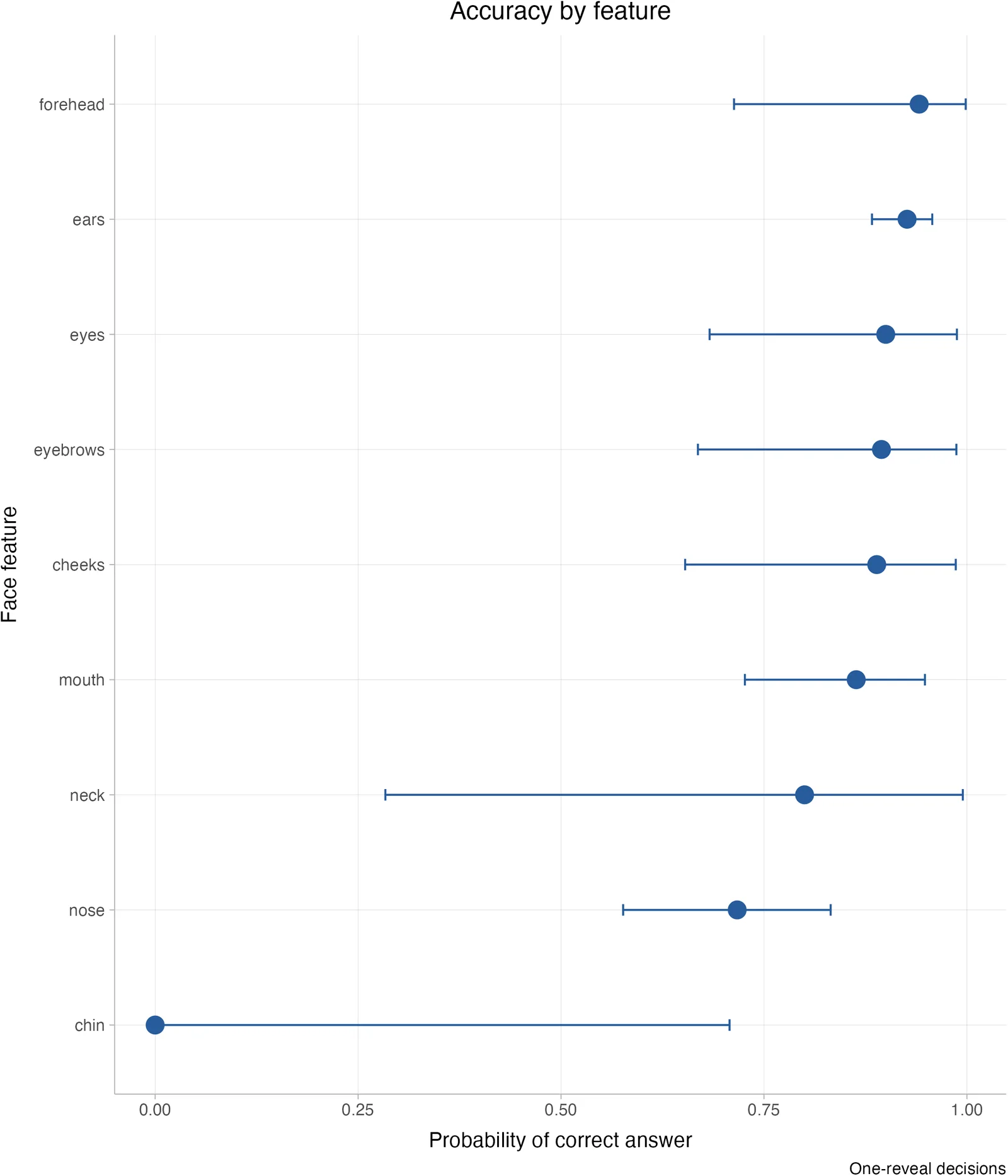
Probability of correct decisions for one-reveal trials by feature revealed. The plot shows the probability of a correct answer (x-axis) based on an exact binomial test for each facial feature (y-axis) when only one feature was revealed. Error bars represent 95% confidence intervals. Features are ordered by their probability of a correct answer.

Foreheads were associated with the highest accuracy (probability of success = 0.94), though the lower bound of the 95% confidence interval reached a comparatively low value of 0.71. In contrast, ears showed a similarly high probability of success (0.93), but with a tighter confidence interval (lower bound: 0.88).

In general, necks, noses, and chins tended to be less reliable for identification than other features. Although the upper bound for necks was relatively high, the confidence interval was wide. Noses had one of the lowest probability estimates, with an upper bound that was the second lowest among the features (0.83). Chins ranked the lowest, exhibiting both a 0% probability of success and a low confidence interval upper bound of 0.71, which was lower than the success probabilities of all other features. The chin was revealed first in only three trials, and each of these trials resulted in an incorrect answer, which explains the 0% estimate and very wide interval. Conversely, other features exhibited upper confidence bounds near 100%. This indicates that while these features may not be universally diagnostic, they can be highly predictive of identity in specific trials. The width of some confidence intervals reflects the substantial variability in feature utility across different image pairs.

Overall accuracy was the same when specialists selected their own features or when the feature was decided for them. However, on a feature-based level, we do not know whether accuracy, when viewing a specific feature, varies depending on whether a specialists makes the deliberate choice to view it. To answer this, we examined trials in which participants correctly identified the probe image with only one feature revealed. We looked at the percentage of times each feature was the one shown. We found that in the self-selected condition ears dominated correct decisions (74.32% of trials). The second most used feature among correct trials with one feature revealed was the nose, corresponding to only 9.46% of trials.

For the predetermined condition, when different features were shown first, a similar pattern emerged. Ears were most often the only feature revealed on correct trials, but with less dominance than in the self-selected condition (34.67% of trials). Mouths and noses were the next most shown first, making up 18.67% and 14.67% of trials, respectively. Therefore, examiners are capable of adapting to the present information at hand, even when it is not their preferred feature.

This analysis focuses on the features associated with successful identifications. A detailed breakdown of the feature combinations revealed during incorrect decisions is provided in the Supporting information (Fig D), showing the patterns of self-selected feature use when identifications were incorrect.

#### Feature preferences.

To gain insight into specialists’ preferences, we examined the self-selection condition. Within this condition, we assessed how frequently participants revealed each feature. Our results indicated that specialists predominantly selected ears (see Fig 7). On average, participants chose their most frequently used feature for 54.26% of their total reveals (*SD* = 14.61%). The total number of features revealed by any participant throughout the experiment ranged from 4 to 9. The preference for ears among specialists aligns with the training and instructions many receive on the job, which often emphasizes identity information present in ears. These findings suggest that specialists leverage this knowledge effectively in their decision-making processes.

**Fig 7:**
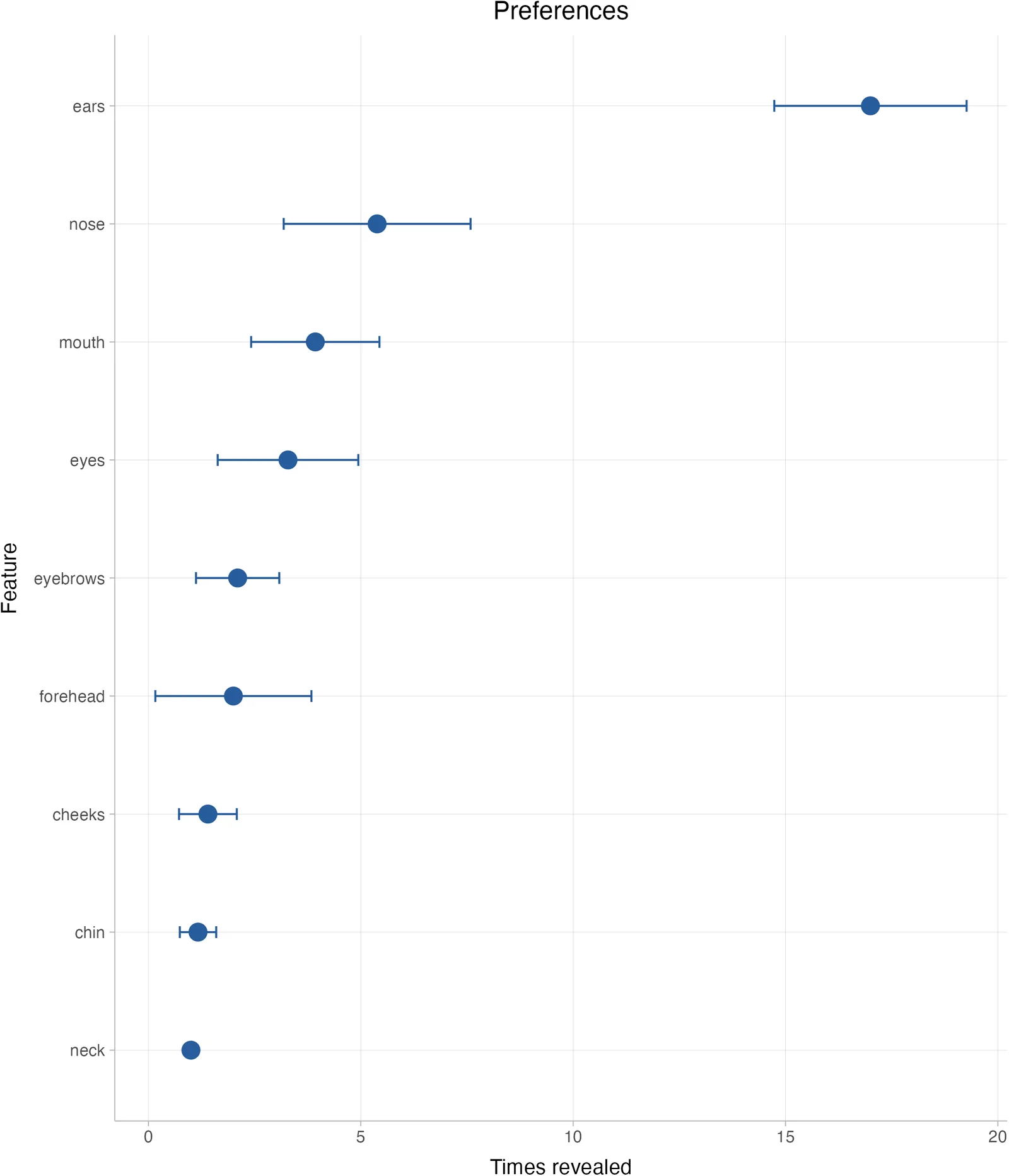
Feature reveal preferences in the self-selection condition. The plot shows the number of times each facial feature (y-axis) was revealed (x-axis) in the self-selection condition, averaged over participants. Features are ordered by their average reveal frequency. Error bars represent the 95% confidence intervals. S2 Table in S1 File in the Supporting information shows the average number of reveals per feature per condition.

To delve deeper into feature selections, we examined which features were utilized, and what factors influenced their selection. We hypothesized that the perceived inter-identity similarity of the visible features in the target images influenced selections. Specifically, we anticipated that features which appeared more dissimilar in the target images would be revealed more often in the probe image than those that were more similar. To investigate this, we calculated the average inter-identity similarity rating for each feature in the benchmarking study, as well as the frequency at which each feature was revealed in the current experiment. By computing the correlation between these two metrics, we found support for our hypothesis that inter-identity similarity influences feature selection. As illustrated in Fig 8, while ears emerged as the most frequently selected feature, features rated as more similar across identities were utilized less often. This suggests a nuanced pattern of behavior: specialists often rely on the same feature (ears) throughout most trials. However, when they do make different selections, these choices are influenced by the similarity of the features. This indicates that specialists possess the flexibility to adapt their selections when necessary.

**Fig 8:**
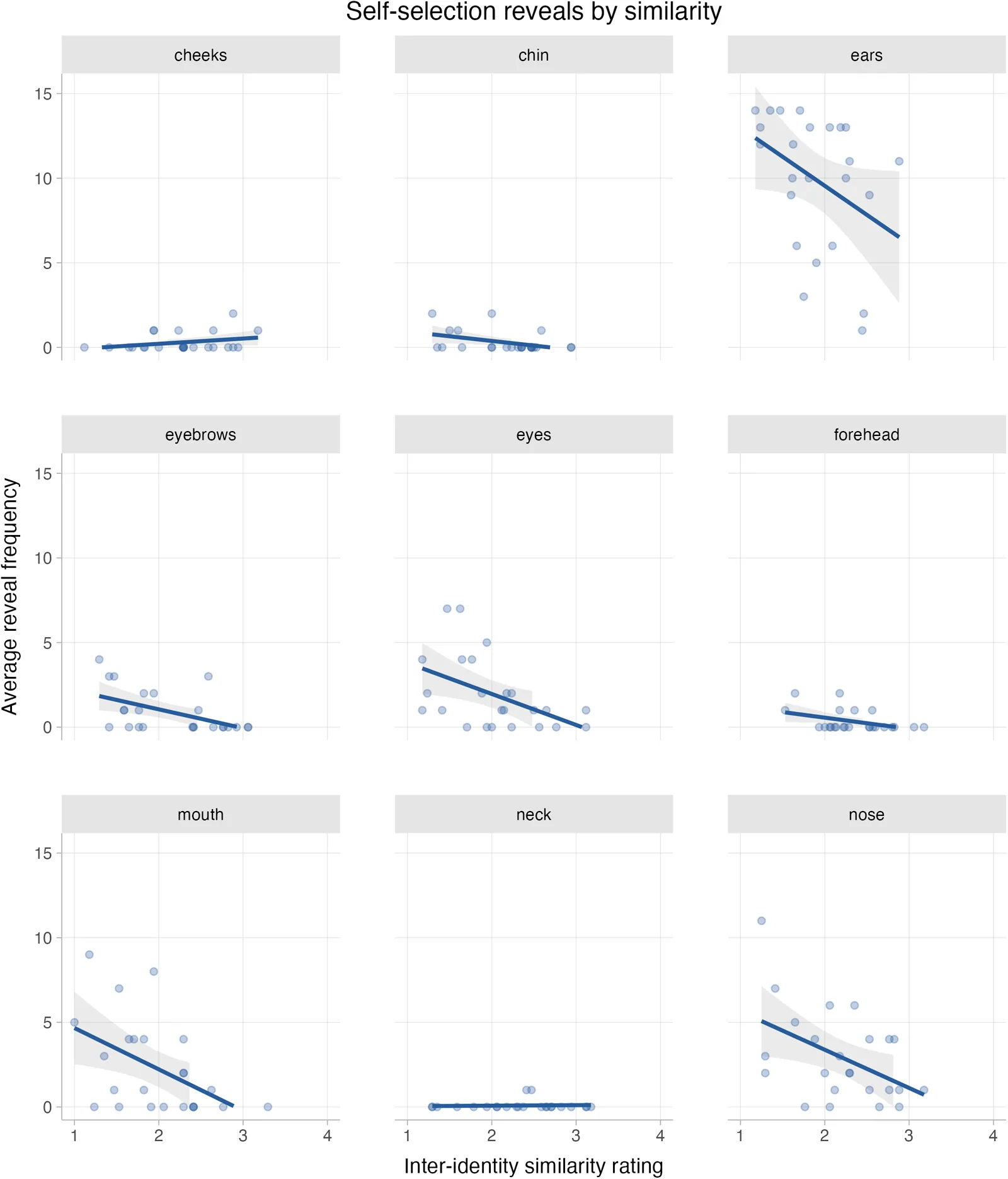
Relationship between facial feature similarity and reveal frequency. The plot shows the average number of times each facial feature was revealed per trial (y-axis) as a function of the rated inter-identity similarity of the target images (x-axis) in the self-selection condition only. Each panel represents a different facial feature. A linear trend line with 95% confidence interval is shown.

#### Perceptual sensitivity.

We next examined the relationship between specialists’ preferences and the features associated with accurate identification decisions. By evaluating these together, we can gain insights into the perceptual sensitivity of experts: does their perception of what is most dissimilar align with the feature’s actual diagnostic utility? To test this, we evaluated trials in the self-selection condition where only a single feature was revealed before a decision was made. Our findings indicate that there is no consistent relationship between the features most frequently chosen first by specialists and the features most strongly associated with a correct answer across all trials. Fig 9 shows this by comparing the ranked order of feature use (preferences for the first reveal) and accuracy (utility of the first reveal). To quantify this, we calculated the Pearson’s r correlation between the average number of times a feature was revealed and the probability of success (r(7) = 0.24, p = 0.53).

**Fig 9:**
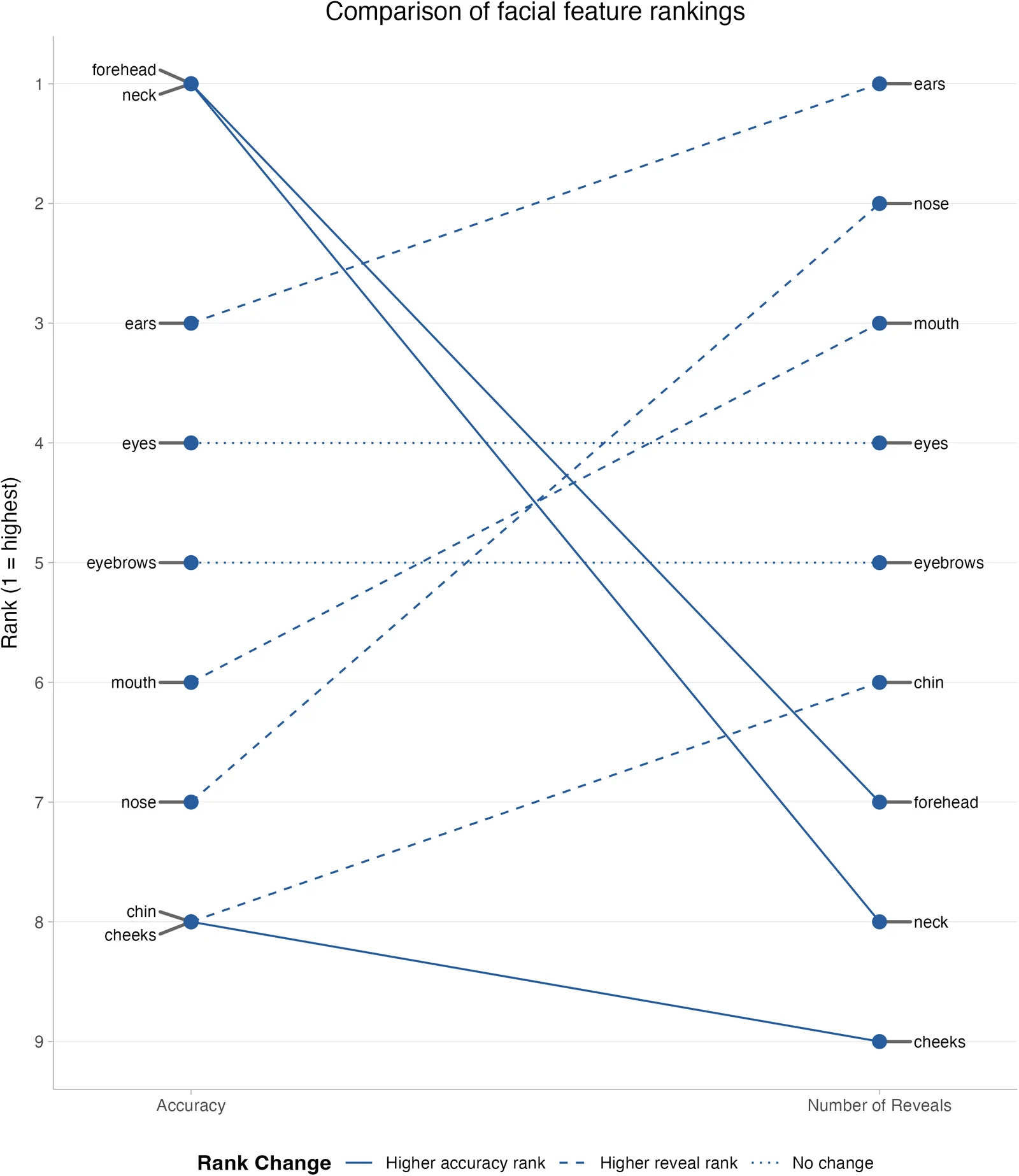
Comparison of facial feature rankings by accuracy and reveal frequency. The slope chart shows the ranking of facial features by their accuracy (left) and the ranking of number of times it was revealed (right) on trials with one reveal. Lines connect the ranks of each feature across the two rankings, with line type indicating the direction of rank change.

However, it is notable that ears were both heavily used and resulted in high accuracy. Therefore, this suggests that ears are likely appropriately used, but for other features, the relationship between feature preferences and accuracy is inconsistent at a group level. This may be a result of specialists not knowing which features are best or it may be because no specific feature is always best to use across different faces.

## Discussion

This study investigated how forensic face specialists utilize various facial features to make identification decisions. Our findings provide valuable insights into the decision-making processes of these specialists, highlighting the importance of certain facial features, such as foreheads, ears, eyes, eyebrows, and cheeks, in accurate identifications. By examining how specialists prioritize different facial features and the extent to which these features lead to accurate decisions, we can better understand the processes underlying their expertise. This understanding supports the development of effective training, protocols, and guidelines for forensic face specialists, ultimately enhancing the accuracy and reliability of facial identification in forensic contexts. Our findings reveal that specialists can be highly efficient and adaptable, requiring little information from the face to be highly accurate, and that they can adapt to evaluating the features available, even when those features may not be their preferred ones.

### Efficiency and feature utility

A key finding is that specialists often require minimal information to reach accurate decisions, often needing to see only one or two facial features. This observation is consistent with findings from [24], which demonstrated that highly accurate face recognizers generally require less visual information than novices to achieve accurate results. While the present study did not include a novice control group, the high efficiency observed among specialists provides converging evidence that expert-level proficiency in facial image comparison is associated with the ability to reach accurate decisions using limited visual information.The limited information they need suggests that while holistic processing is the default mode for human face perception and recognition [8], it is not strictly required to conduct an accurate match. When a feature is sufficiently diagnostic, even when viewed in isolation, specialists can successfully extract identity information from it. The finding that participants frequently achieved high accuracy after only a single reveal — despite having the option to reveal more — underscores the high diagnostic potential of features in isolation. Importantly, the choice to reveal subsequent features after the first seems to be driven by a mechanism of self-regulated accuracy. In the “Similar first” condition, participants were more likely to reveal a second feature compared to the “Dissimilar First” condition. This suggests that specialists possess an internal gauge of sufficiency. Because participants could choose to reveal as many or as few features as they felt were necessary to reach a correct decision, the increased reveal rate in the “Similar first” condition indicates that these trials more frequently required additional evidence to reach that threshold. Thus, while the probability of accuracy remained comparable across conditions, the evidence-gathering process was more extensive when initial features were more similar across identities, demonstrating a self-regulated approach to reaching a decision. Consistent with this, the absence of a group-level correspondence between feature preferences and accuracy suggests that specialists leverage a general starting preference but rely on a dynamic, case-by-case evaluation to determine when further visual evidence is required to reach an accurate decision.

Interestingly, the probability of a correct response did not monotonically increase as more features were revealed; in the self-selection condition, the average probability decreased after three reveals, and confidence intervals widened. This pattern suggests that specialists may not be fully aggregating and integrating information across all revealed features. Instead, they may be primarily relying on the most recently revealed feature to make their decision. Because previously revealed features remained visible on the screen, this is unlikely to be a failure of working memory. Rather, it may reflect a cognitive tendency to prioritize recent evidence over a collective set of features. In professional practice, examiners typically utilize workbooks, checklists, or detailed notes to systematically accrue and weight evidence across the entire face. The absence of such aides in the current experimental design may have limited the participants’ ability to effectively aggregate information.

Our results are consistent with previous research showing that certain facial features are more diagnostic than others for face recognition [7,12]. While no single feature is universally diagnostic for all facial comparisons [14], our findings reveal general trends in feature-based accuracy across the images used in this study, despite large variability in the inter-identity similarity ratings of individual features. Previous research by [16] and [12] indicated that examining specific facial parts enhances accuracy in matching; however, their studies focused on features within the context of the entire face. Because holistic face processing can hinder the identification of distinct facial parts [9], it remains an open question whether face matching should focus on specific facial features rather than the complete face. Future research should explore this notion in more detail in light of our findings which demonstrate that many of the same features found to be useful when viewing whole faces remain valuable for matching even when viewed in isolation.

### Expert strategies and adaptability

Their adaptability suggests a degree of perceptual alignment regarding the visual information from the face. When the visible features appeared more similar in the images, those features were less often selected when specialists had a choice of which feature to reveal. Similarly, specialists were more likely to reveal more parts of the face when the features visible were judged to be similar. This indicates that specialists use the perceived dissimilarity of features to guide their selection process. While this selection increases efficiency by reducing the number of reveals needed, it is not the only path to accuracy; the high adaptability observed suggests that specialists can compensate for a non-diagnostic first reveal by gathering additional evidence. However, there was a misalignment between perceived and actual utility for some features. While specialists correctly prioritized the ears (which yielded high accuracy), they consistently undervalued the forehead. In single-feature trials, the forehead was associated with the highest probability of a correct decision, yet it was rarely selected voluntarily. We note that because the forehead was selected so infrequently, the confidence intervals regarding its success probability are wide; however this finding suggests that specialists may lack either the awareness of or perceptual sensitivity to the diagnostic power of the forehead, including the upper face and hairline. Conversely, central features like the nose and mouth were selected moderately often but yielded lower accuracy. This mirrors findings by Dunn et al. [12], where participants improved when guided to useful features but could not identify them independently. A more definitive study comparing matching accuracy when viewing foreheads versus other parts of the face, such as ears, is needed to form stronger conclusions. However, this work shows that there are potentially undervalued areas of the face that may be useful for identification.

### Implications for forensic practice

This carries significant implications for training specialists. Given that certain features (such as foreheads and ears) display greater predictive value for accurate decisions, it may be beneficial to incorporate information regarding the relative diagnostic utility of these features into existing guidelines. We do not suggest that these findings should replace the comprehensive morphological analysis required for a full forensic examination, where a comparison of all available features is standard practice. However, emphasizing the importance of highly diagnostic features may be particularly beneficial in high-throughput assessment or review scenarios where speed is a critical factor. Furthermore, for full examinations, awareness of these high-utility features could serve as a secondary check to ensure that the most diagnostic information has been fully considered before a final conclusion is reached.

Beyond training human specialists, these findings have implications for the field of human and artificial intelligence collaboration. Just as crowd-sourcing has been used to train algorithms on diagnostic regions in bird classification [21], it is feasible that facial specialists could significantly contribute to forensic contexts by assisting in training face recognition algorithms. Existing literature indicates that combining human and algorithmic decision-making yields more accurate outcomes than either approach alone [1]. Studies have already begun to examine how skilled individuals can improve face matching algorithms, demonstrating that visual fixations from experts can enhance algorithmic accuracy [25] and that visual information sampled from viewing patterns face super-recognizers improved identity matching accuracy in a set of deep neural networks [26]. Future research should explore how trained face expert-defined diagnostic features can further enhance the reliability of face recognition algorithms.

An unresolved question is whether specialists would rely on different features in a two-alternative forced-choice task where they are required to judge face pairs as the same or different. This could be critical, as specialists focus on criteria for both exclusions (determining that two images are of different people) and inclusions (determining that two images are of the same person). Future research should aim to identify which features, if any, are more helpful for matching individuals together versus distinguishing between them.

Another potential factor influencing decisions could be the unexpected appearance of a feature in the probe image. Inter-identity feature similarity was assessed based on ratings comparing two visible target images. The database used for the study deliberately contained image variability, incorporating differences in expressions, lighting, facial hair, glasses, and more. Consequently, the facial features that were judged to be different between the two target images might not be the most effective for comparing to the probe image. Nonetheless, specialists adapted, achieving high accuracy in all conditions, even with only one or two features shown.

### Limitations and future directions

The study has several limitations worth noting. Firstly, it is essential to understand that the findings do not accurately reflect real-world accuracy, as the conditions under which the study was conducted were artificial and specifically designed to address certain research questions. Furthermore, the features analyzed in this research may not generalize well to different sets of images. Our findings are based on 40 image triads subselected from the TIM test using criteria designed to ensure the task was challenging. This could result in the identification of diagnostic markers that might not be as prominent in cases where comparisons are not based on a subsample of difficult trials. Additionally, this specific set may possess properties based on the particular camera and photograph conditions of this study not found in other collections. For instance, eyes were often depicted as relatively small in this study, which may not hold true across other facial imagery. Another limitation arose from the demographic composition of the images which primarily included individuals who were Caucasian and East Asian. Consequently, the facial features examined may not represent those that are useful for facial comparisons across diverse populations. Testing a wider range of stimuli types will also be needed to form generalized conclusions, especially including stimuli types that are often encountered by specialists such as comparing security-camera footage stills to a mugshot, comparing across poses, lighting conditions, and other factors known to impact matching accuracy in other studies [27–29]. Additionally, because this study did not include a novice control group, these findings are specific to trained specialists and may not generalize to the general population, who may not possess the same trained sensitivity to diagnostic features such as the ears. Lastly, it is critical to recognize that the features discussed in this study are not exhaustive. The research focused on those features that could be isolated automatically and that are prevalent across nearly all human faces globally, limiting the scope of the analysis.

Additionally, our use of pre-defined feature boundaries may have overlooked diagnostic details that traverse these regions, such as specific creases, marks, or scars that cross from one feature area to another. While the boundaries were developed in consultation with experts to ensure consistency across participants, this rigid compartmentalization differs from real-world examinations where specialists evaluate the face holistically. This represents a limitation of the experimental design, although the inability of participants to zoom into images likely mitigated the impact of such boundary-crossing details.

Building on these results, future research could use a controlled reveal design to better understand how specific combinations of features contribute to accuracy. While our experimental design permitted the reveal of multiple features, the specialists in this study were highly efficient and predominantly reached decisions after only one or two reveals. This provided insufficient data to analyze the impact of feature combinations. Future research could employ a forced-reveal design to specifically examine whether the spatial arrangement of features (e.g., clusters of adjacent features versus widely spread features) differentially impacts accuracy. Exploring these relationships further will help the field develop a more comprehensive understanding of best practices for forensic face experts. Additionally, it would be valuable to collect confidence scores for decision-making in future studies. Comparing confidence levels between self-selected and predetermined feature reveals could reveal whether specialists’ internal gauge of sufficiency (how many features to reveal) aligns with their subjective confidence in the resulting decision.

Conducting this research with scenarios more closely matched to forensic workflows is a promising direction for future exploration. Forensic specialists are not merely following a static script. They are efficient decision-makers who adapt their strategies based on the visual information available. While professional roles vary, ranging from high-throughput reviewers to forensic examiners, these specialists share a foundation of specialized training. Examiners typically receive more extensive training than reviewers, including instruction in the use of formal facial feature checklists, but both groups are generally trained to attend to specific face regions: its shape, eyes, ears, nose, mouth, and chin and jaw [30]. This shared foundational knowledge likely informs how specialists prioritize features. By refining guidelines to highlight the relative diagnostic value of specific features, the field can support specialists in adapting their strategies to the specific requirements of the task, whether conducting a rapid assessment or a comprehensive forensic examination, thereby enhancing the overall reliability of facial identification.

## Supporting information

S1 File Supplemental figures, tables, analysis, and feature boundary details. This file contains supplemental figures comparing testing methods (remote vs. in-person), tables of feature reveal frequencies by condition and accuracy by feature revealed, binomial test results for feature accuracy, and frequency of feature combinations. It also includes detailed descriptions of the machine learning landmarks used to define facial feature boundaries (Eyes, Eyebrows, Nose, Mouth, Chin, Cheeks, Forehead, Ears, and Neck).
(PDF)
