# Supplementary material for: Facial feature prioritization by forensic specialists
Source: PLoS One. 2026 Oct 21;21(10):e0359520. doi: 10.1371/journal.pone.0359520 (PMC13686473; doi:10.1371/journal.pone.0359520)
Supplement: Supplementary file 1 [file pone.0359520.s001.pdf]

Supporting Information: Facial feature prioritization by forensic specialists

Carina A. Hahn, P. Jonathon Phillips, John D. Grantham, Amy N. Yates

A Supplemental Figures

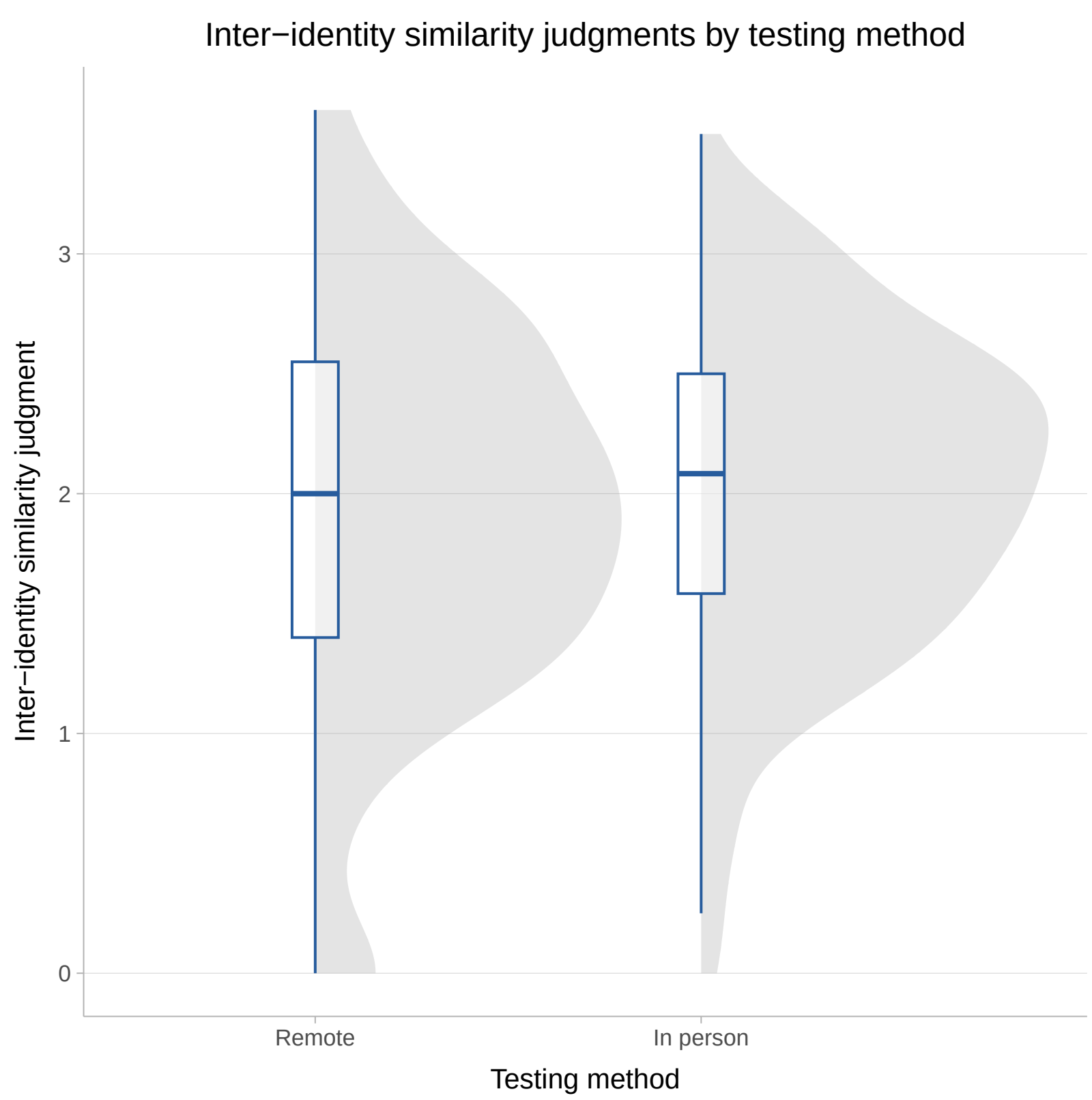

Figure A. Comparison of inter-identity similarity judgments between remote and in-person participant samples. The plot shows the distribution of inter-identity similarity judgments (y-axis) for remote and in-person participant samples (x-axis). Density plots illustrate the distribution of judgments, while box-and-whisker plots indicate the median and interquartile ranges.

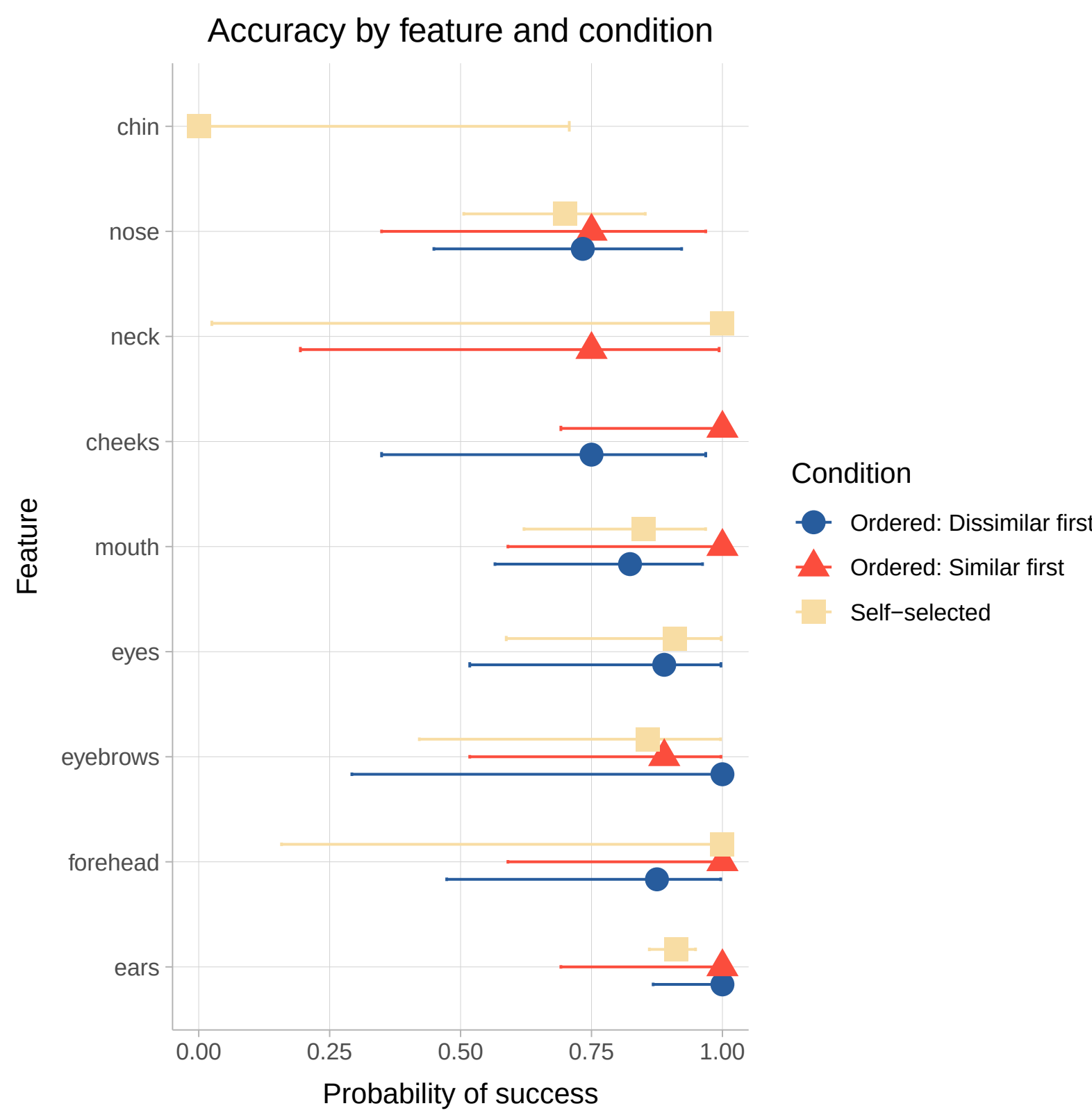

Figure B. Accuracy of one-reveal decisions by facial feature and condition. The plot shows the probability of a correct answer based on an exact binomial test (x-axis) for each facial feature (y-axis) and experimental condition for trials in which decisions were made after one feature revealed. Error bars represent 95% confidence intervals.

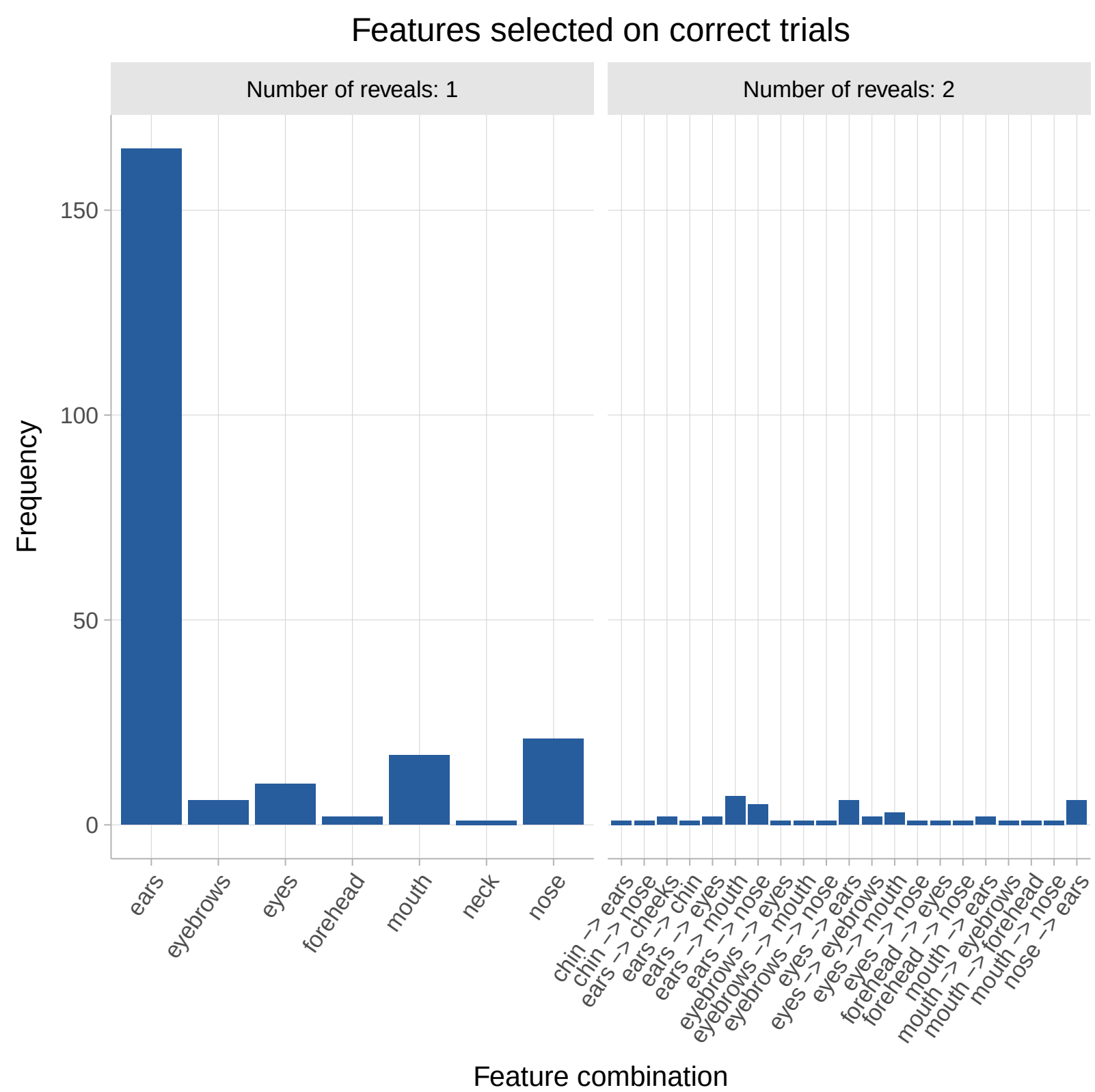

Figure C. Features revealed on correct trials. Features revealed on correct trials in the self-selection condition. The plot shows the frequency of different feature combinations for trials with one or two reveals.

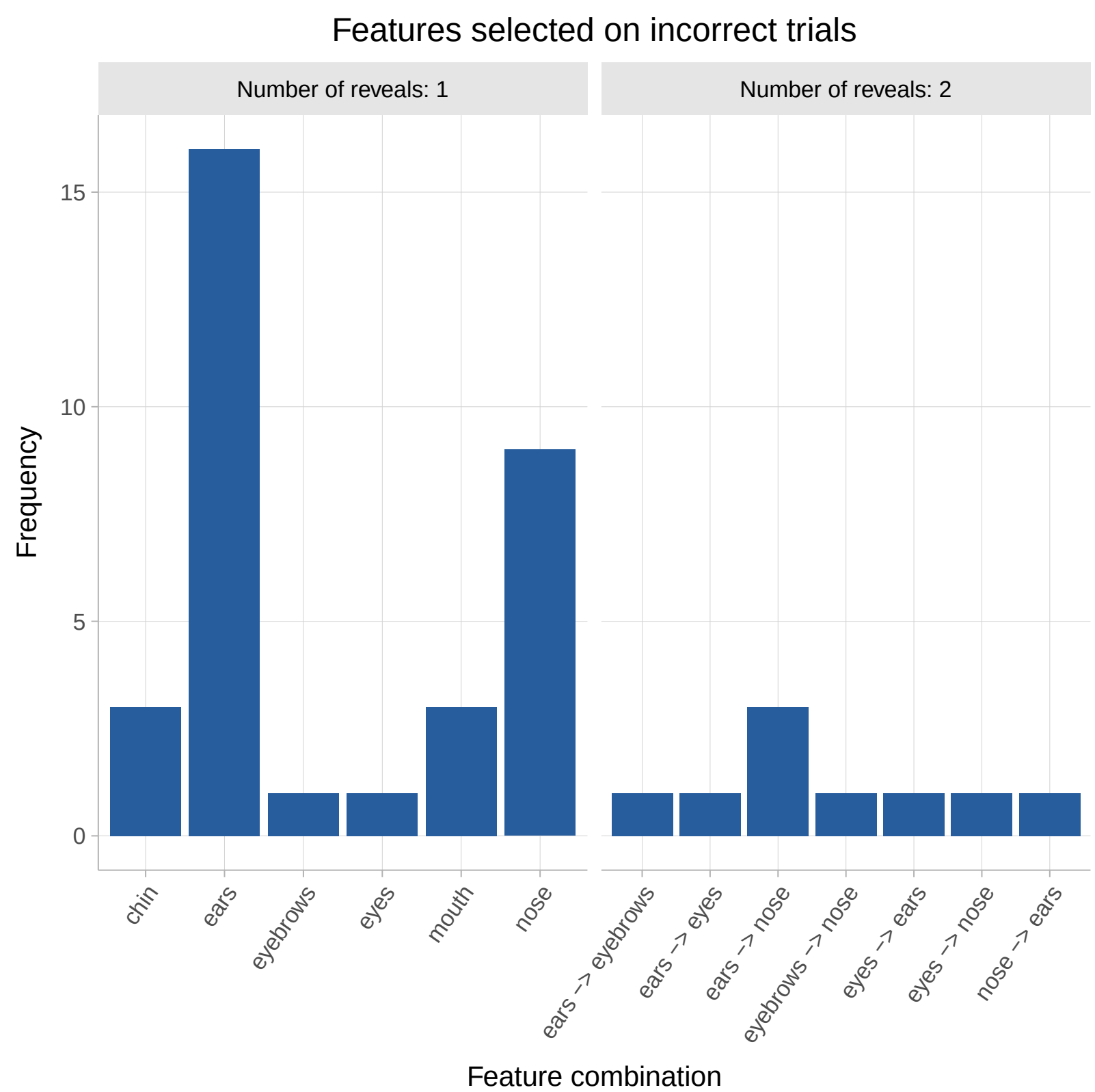

Figure D. Features revealed on incorrect trials. Features revealed on incorrect trials in the self-selection condition. The plot shows the frequency of different feature combinations for trials with one or two reveals.

B Supplemental Tables

Table S1. Accuracy by feature.

Facial feature	Success Probability	95% Confidence Interval
forehead	0.94	[0.71, 1.00]
ears	0.93	[0.88, 0.96]
eyes	0.90	[0.68, 0.99]
eyebrows	0.89	[0.67, 0.99]
cheeks	0.89	[0.65, 0.99]
mouth	0.86	[0.73, 0.95]
neck	0.80	[0.28, 0.99]
nose	0.72	[0.58, 0.83]
chin	0.00	[0.00, 0.71]

Probability for a correct answer on trials where decisions were made with only one reveal. 95% Confidence intervals (CI) are shown as [lower CI, upper CI].

Table S2. Feature reveal frequency.

Facial feature	Self-selected	Ordered: Dissimilar first	Ordered: Similar first
ears	12.93 [10.80, 15.06]	2.00 [2.00, 2.00]	1.00 [1.00, 1.00]
eyebrows	1.40 [0.29, 2.51]	1.00 [1.00, 1.00]	1.29 [0.83, 1.74]
eyes	1.22 [0.71, 1.73]	1.80 [1.24, 2.36]	-
mouth	2.00 [0.99, 3.01]	2.12 [1.30, 2.95]	1.75 [0.23, 3.27]
nose	2.73 [1.56, 3.89]	1.36 [1.02, 1.70]	1.00 [1.00, 1.00]
cheeks	-	1.00 [1.00, 1.00]	1.25 [0.86, 1.64]
forehead	2.00 [-]	1.00 [1.00, 1.00]	1.00 [1.00, 1.00]
chin	1.00 [1.00, 1.00]	-	-
neck	1.00 [-]	-	1.00 [1.00, 1.00]

Mean [95% CI] number of times each feature was revealed per condition on trials where decisions were made after one reveal, averaged by participant. Values shown as Mean [lower CI, upper CI]; “-” denotes the feature was not revealed.

C Feature combination preferences

We looked at whether specific combinations of features resulted in different levels of accuracy. For this, we looked only at the self-selection condition (Figs C and D).

Although there is a consistent preference for ears alone, there is not a strong, shared preference once more than one feature is revealed. Due to the small number of instances in which participants selected more than one feature, it is currently unreliable to assess the relationship between combinations of features and accuracy. There are only 56 trials in which participants revealed two features out of a total of 336 trials; in only 25 trials total did participants select 3 or more features.

D Feature boundary details

We used the dlib machine learning library to identify landmarks and then created feature masks using `cv2.fillPoly`.

1. **Eyes (Left and Right)**: Each eye mask includes the area from the eyelid (midpoint between eyebrow and upper eye edge) down to a line halfway between the lower eye edge and nose bridge. The mask includes the entire eye region, not just the eye itself.

2. **Eyebrows (Left and Right)**: Each eyebrow mask covers the area from the eyebrow landmarks down to the eyelid (midpoint between eyebrow and eye). This includes the eyebrow and the skin between the eyebrow and upper eyelid.

3. **Nose**: The nose mask extends from between the eyebrows down to the nostrils, and horizontally from the inner corner of one eye to the other. The mask is widened at the bottom to include the sides of the nose extending toward the lips.

4. **Mouth**: The mouth region includes the area from the bottom of the nose down to a line halfway between the lower lip and jawline.

5. **Chin**: The chin mask covers the area from the halfway point between lower lip and jawline down to the jawline itself. It spans horizontally across the lower part of the face.

6. **Cheeks (Left and Right)**: Each cheek mask covers the side area of the face bounded by the eye, ear, nose, mouth, and jawline. The left cheek extends from the left

side of the face to the nose and left eye, while the right cheek extends from the right side to the nose and right eye.

7. **Forehead**: The forehead mask covers the area from the top of the image down to the eyebrows, spanning the entire width of the face from the leftmost to rightmost facial landmarks.

8. **Ears (Left and Right)**: The ear masks cover the areas outside the facial landmarks on each side. The left ear mask extends from the left edge of the image to the left side facial landmarks, while the right ear mask extends from the right side facial landmarks to the right edge of the image.

9. **Neck**: The neck mask covers the area from the jawline down to the bottom of the image, spanning the entire width of the lower face.
