# Supplementary material for: Facial feature prioritization by forensic specialists
Source: PLoS One. 2026 Oct 21;21(10):e0359520. doi: 10.1371/journal.pone.0359520 (PMC13686473; doi:10.1371/journal.pone.0359520)
Supplement: Supplementary file 2 [file pone.0359520.s002.pdf]

Dear Dr. Wilkinson,

Thank you for giving us the opportunity to submit a revised draft of our manuscript, "Facial Feature Prioritization by Forensic Specialists." We are grateful to you and the reviewers for the time and effort dedicated to providing feedback, and we appreciate the insightful comments and valuable suggestions that have substantially strengthened the paper.

We have incorporated the suggestions made by the reviewers and provide a detailed point-by-point response below. Those changes are highlighted within the manuscript (tracked-changes version included with this submission). We labeled each reviewer point according to reviewer and comment number (e.g., R1-P1). All page numbers refer to the revised manuscript file with tracked changes.

Reviewers' Comments to the Authors:

Reviewer 1

The rationale of this study is clear and it addresses an interesting and important question. The methods used are novel and mostly well explained. I am convinced by the findings as they relate to accuracy, adaptability, and efficiency, but I'm not entirely sure of the logic regarding the findings about metacognition on lines 340-355 (Figure 9). It is clear throughout this study that the most diagnostic feature varies between face pairs.

Reviewer 1-Point 1 (R1-P1): If there was a relationship across trials between the feature most often chosen first and the feature most often leading to a correct response after the first selection, the interpretation might be that features are equally diagnostic across different pairs of faces (but I'm also not sure, based on the construction of the sentences here, whether this is accuracy amongst only first-feature reveals or all trials).

Author response: Thank you for this clarification. We have updated the text to specify that these results pertain to single-reveal trials in the self-selection condition, and clarified the interpretation accordingly. Because no significant correlation emerged between the features most frequently chosen first and those most often associated with a correct response, the diagnostic utility of facial features varies across image pairs. Because participants could reveal additional features when the first selection proved insufficient, the results further suggest that specialists apply a general starting preference but adjust their evidence-gathering on a case-by-case basis. These changes appear in the Discussion, "Efficiency and feature utility" subsection (p. 18).

R1-P2: The ability to pick the most diagnostic feature also may not be metacognitive in nature but reflect actual sensitivity to the similarity of features – the examiner may not have been able to identify perceptually what the most dissimilar features were, rather than not having insight into this or the fact that it might help them to make a more efficient decision.

Author response: The reviewer is correct that the ability to prioritize features may reflect sensitivity to feature similarity rather than a conscious metacognitive process, and we agree that our original framing conflated these concepts. Accordingly, throughout the manuscript we have replaced the term "metacognition" with "perceptual sensitivity," ensuring the interpretation remains closely aligned with the data and avoids inferences about processes that were not directly tested. The revised subsection heading ("Perceptual sensitivity," formerly "Metacognition") and the rewritten paragraph appear in the Results, "Perceptual sensitivity" subsection (p. 16). Related language in the Introduction (p. 3) and Discussion has also been updated (p. 20).

R1-P3: The ability to select a different feature than the one that would be most useful and still answer correctly seems to be central to this adaptability that the authors also focus on and make a convincing argument for. How important do the authors think that metacognitive ability in selecting which features to focus on is when the ability to adapt is so high – does one need to focus on the most diagnostic features if they can perform the task equally well without doing this?

Author response: We think this is an excellent question. Although specialists are highly adaptable, as shown by their gathering of additional evidence when the first feature is not diagnostic, prioritizing diagnostic features remains critical for efficiency. By selecting the most dissimilar features first, specialists reduce the number of reveals needed to reach a correct decision. The updated manuscript emphasizes that while adaptability ensures accuracy, perceptual sensitivity to diagnostic features drives efficiency. These revisions appear in the Discussion, "Expert strategies and adaptability" subsection (p. 20).

R1-P4: One additional minor point from lines 418-420: "We note that because the forehead was selected so infrequently, the confidence intervals regarding its success probability are wide." It would be good to know just how infrequently, considering this feature was reported to be associated with the highest probability of success.

Author response: We agree that the results would benefit from more explicit data on reveal frequency. Accordingly, we added Table S2 to the Supporting Information to complement Fig. 7; the table reports the exact reveal frequency across experimental conditions. We have also updated the caption of Fig. 7 to reference this table. The new table appears in the Supporting Information as Table S2 (SI p. 5). The updated Fig. 7 caption appears in the main text (p. 15).

Reviewer 2

Overall, the manuscript is written and structured well. The authors have provided details of demonstrated rigour with regards to the experimental methods and subsequent analysis. All supporting data is present. The contribution to the field is interesting, and could be useful, but requires some more clarity.

The main body of text is well laid out, with explanations for each stage. The more discursive sections are also well thought out and provide justification for the experimental design and interpretation of results. There are some elements that could be described and clarified further:

R2-P1: In the Introduction you state that, "…face specialists, perform the critical task of comparing facial images to determine identity". This implies a formal identification (match) can be made, even though it cannot. For a full examination specialists will usually provide a level of support for their conclusion rather than just saying it is or isn't the same person. I think this needs adding in for clarification.

Author response: As suggested by the reviewer, we have revised the introductory sentence to clarify that specialists provide a level of support rather than a definitive determination. The line has been revised from "…face specialists, perform the critical task of comparing facial images to determine identity" to "face specialists, perform the critical task of comparing facial images, often indicating how strongly the evidence shows the images are either the same person or different people." This revision appears in the Introduction, paragraph 1 (p. 2).

R2-P2: - The use of occlusion and boundaries for visualising features in isolation is well thought out. However, was there any feedback from participants regarding detail that traversed different boundary sections? For example, perhaps a crease, mark or scar was on the boundary line?

Author response: There were instances in which facial details can extend beyond the boundaries of the defined feature regions. We have added a statement to the "Feature isolation" subsection clarifying that we did not obtain specific feedback on such occurrences, and we have expanded the "Limitations and future directions" subsection to acknowledge that this compartmentalization differs from holistic real-world examinations. We also note that the image resolution and inability to zoom in on the images likely minimized the impact of any boundary-crossing details in the current study. The statement about boundary feedback appears in the Methods, "Feature isolation" subsection (p. 10-11). The expanded limitation appears in the Discussion, "Limitations and future directions" subsection (p. 23).

R2-P3: - I notice in the stimulus example given that the nose boundary cuts through the inner corner of the eye (a useful feature for FIC). Was this the case for all stimuli?

Author response: Thank you for pointing this out. The boundary did cross the inner corner of the eye in other stimuli, though not in all cases. Expert feedback was obtained and the boundaries were approved. The reviewer is correct that forensic examiners evaluate minutiae such as the inner corner of eyes during detailed examinations. Because images were viewed at a fixed resolution in an interface that did not enable zooming, this trade-off was appropriate for the scope of the study. We have added the following clarification in tandem with the edit in response to R2-P2 to the "Feature isolation" subsection (p. 11):

> "We note that we sacrificed some precision in the feature boundaries for the sake of having consistent definitions of the coordinates across participants. Because

> participants would be unable to zoom into images to view minutiae or the small details of the features, we felt this trade-off was appropriate in this study."

R2-P4: - Can you comment on the decision to only occlude the probe image and not the target faces as well?

Author response: As suggested by the reviewer, we have added a sentence to the "Experimental Design and Procedure" subsection explaining this design choice. The new text reads as follows (p. 8):

> "This design allowed specialists to form a full-face assessment of the target identities firsst and then selectively reveal features on the probe to verify their assessments about which specific features would be most diagnostic to reveal on the probe image for that particular pair of target images"

R2-P5: - Additionally, can you comment on the choice to gradually reveal the face, rather than only showing features in isolation?

Author response: The design choice was implemented to allow participants to leverage combinations of features if necessary. We did not anticipate that only one feature would be revealed for the majority of trials. We have clarified the rationale for the gradual reveal design in the Introduction (p. 3). We have also updated the Discussion to emphasize that the high frequency of single-feature reveals underscores the diagnostic potential of individual features. The new text reads as follows (p. 18):

> "The finding that participants frequently achieved high accuracy after only a singe reveal – despite having the option to reveal more – underscores the high diagnostic potential of features in isolation."

R2-P6: 27 participants were recruited with face specialist experience and/or training. Was any data collected on the level of experience? E.g. through number of years of practice, or through self-assessment of experience (e.g. novice, very experienced... etc.). What type of facial image comparison were they conducting? Full examinations, or reviews, or assessments?

Author response: This point is addressed in conjunction with Reviewer 3, Point 12. The "Participants" section now explicitly states that specific professional roles and years of experience were not collected (p. 11). The "Limitations and future directions" subsection has also been expanded to acknowledge differences in training between examiners and reviewers, while noting that the shared foundational training in facial regions makes these distinctions secondary to the goals of the current study (p. 24):

> "While professional roles vary, ranging from high-throughput reviewers to forensic examiners, these specialists share a foundation of specialized training. Examiners typically receive more extenstive training than reviewers, including instruction in the use of formal facial feature checklists, but both groups are generally trained to attend to specific face regions: its shape, ears, nose, mouth, and chin and jaw

[31]. This shared foundational knowledge likely informs how specialits prioritize features."

[31] Moreton R, Havard C, Strathie A, Pike G. An International Survey of Applied Face-Matching Training Courses. Forensic Science International. 2021:;327:110947. doi: 10.1016/j.forsciint.2021.110947.

R2-P7: - It would have been interesting to collect confidence scores for their decision making. Perhaps the self-selected trials would have yielded different confidence scores to the predetermined ones.

Author response: Thank you for this suggestion. It would indeed be valuable to collect confidence scores to examine whether specialists' internal gauge of sufficiency aligns with their subjective certainty. We have added to the "Limitations and future directions" subsection recommending that future studies collect confidence measures. The revised text reads as follows (p. 24):

> "Additionally, it would be valuable to collect confidence scores for decision-making in future studies. Comparing confidence levels between self-selected and predetermined feature reveals could reveal whether specialists' internal gauge of sufficiency (how many features to reveal) aligns with their subjective confidence in the resulting decision."

R2-P8: The discussion could include sub-headings to break up the text.

Author response: As suggested by the reviewer, we have added the following subsections to the Discussion:

- Efficiency and feature utility
- Expert strategies and adaptability
- Implications for forensic practice
- Limitations and future directions

Content has been redistributed across these subheadings and some transitional sentences edited, but the substance is unchanged except where other reviewer comments required revision.

R2-P9: - In the discussion, you say that "...it may be beneficial to incorporate recommendations for evaluating diagnostic features into existing guidelines". The FISWG feature list currently outlines all morphological detail to be examined. Are you suggesting to direct examiners to prioritise areas with high diagnostic value or just cueing them to look at them? (The feature list already does the latter).

Author response: We agree that the current FISWG guidelines already cue examiners to an extensive list of morphological details. Our work complements this list by providing empirical evidence on the relative diagnostic utility of those features. Knowing which features are

most predictive of identity can help specialists prioritize their attention, particularly where time and efficiency are critical. The "Implications for forensic practice" subsection has been updated to specify that we are suggesting a prioritization of attention, not a replacement for the comprehensive feature list. This revision appears in the Discussion, "Implications for forensic practice" subsection (p. 21). This revision also incorporates edits in response to R2-P10 and R2-P11.

R2-P10: - There needs to be some clarity on which processes you are referring to (assessment, review, examination) as they have different requirements and uses (you mention both examiners and reviewers as Face Specialists). Utilising the most diagnostic features for a FIC seems sensible, but it seems unlikely that this would result in only looking at certain features, and not all of them, specifically in an examination scenario. An FIC examination report would include comparison of all features, not just some. Are you suggesting that full examinations could be completed using only relevant diagnostic features? Or are you suggesting this for an assessment or review rather than an examination?

Author response: The reviewer draws a critical distinction between the procedural requirements of a formal examination and the cognitive process of identification — a distinction we agree is important. A full forensic examination report must include a comprehensive comparison of all available features to meet professional and legal standards, and our findings do not challenge this. Rather, we describe the behavioral efficiency with which specialists reach decisions. The Discussion, "Implications for forensic practice" has been updated to distinguish explicitly between these two contexts. The added text reads as follows (p. 21):

> "We do not suggest that these findings should replace the comprehensive morphological analysis required for a full forensic examination, where a comparison of all available features is standard practice. However, emphasizing the importance of highly diagnostic features may be particularly beneficial in high-throughput assessment or review scenarios where speed is a critical factor. Furthermore, for full examinations, awareness of these high-utility features could serve as a secondary check to ensure that the most diagnostic information has been fully considered before a final conclusion is reached."

R2-P11: - Based on the above comment, given that one feature has been shown to provide enough information for a conclusion, its benefit is in the time it has taken, compared to a full morphological comparison of the whole face (examination) that would take longer. It seems that this would be of benefit to facial assessment processes used in scenarios where speed is required (e.g. border control).

Author response: We agree that efficiently identifying diagnostic features is particularly valuable in high-throughput scenarios such as border control, where speed is a priority. The Discussion has been updated to highlight such assessment contexts as a primary application for our findings, while maintaining the distinction that this does not supersede the requirements for a comprehensive forensic examination. This update appears in the Discussion, "Implications for forensic practice" subsection (p. 21).

R2-P12: I think the suggestion of future research that enables the analysis of features used past the point of the first feature would be useful. It might be worth adding more to this regarding how the combination of features used might impact accuracy – e.g. if the features are side by side, or if they are spread.

Author response: Thank you for this suggestion. We have expanded the "Limitations and Future Directions" section to acknowledge that while our design permitted multiple reveals, specialists' efficiency resulted in too few multi-feature trials to analyze combinations. We explicitly suggest that future research use a forced-reveal design to examine whether the spatial arrangement of features — adjacent versus spread — affects accuracy. This addition appears in the Discussion, "Limitations and future directions" subsection (p. 23-24):

> "While our experimental design permitted the reveal of multiple features, the specialists in this study were highly efficient and predominatnly reached decisions after only or two reveals. This provided insufficient data to analyze the impact of feature combinations. Future research could employ a forced-reveal design to specifically examine whther the spatial arrangement of features (e.g., clusters of adjacent features versus widely spread features) differentially impacts accuracy."

Reviewer 3

This manuscript provides an assessment of facial feature selection and utility by trained face specialists, and is a welcome and important addition to the literature. I enjoyed reading the manuscript, particularly your approach using facial image triads, as well as the feature revealing process.

R3-P1: There are some general limitations to the study that I feel need to be highlighted and addressed, namely: The small set of stimuli limits the generalisability of the results to other types/sources of facial imagery, although the utility of the ears is supported by other literature. This limitation is touched upon in the discussion but I think should be reinforced.

Author response: We have reinforced the limitation as discussed by this reviewer. The "Limitations and future directions" section has been expanded to clarify that our findings are based on 40 image triads specifically sub-selected from the TIM test to ensure the task was challenging, and that this deliberate selection combined with specific camera conditions may result in diagnostic markers that are less prominent in other image databases. The expanded limitation appears in the Discussion, "Limitations and future directions" subsection (p. 22-23) to include:

> "Our findings are based on 40 image triads subselected from the TIM test using criteria designed to ensure the task was challenging. This could result in the identification of diagnostic markers that might not be as prominent in cases where comparisons are not based on a subsample of difficult trials. Additionally, this

specific set may possess properties based on the particular camera and photograph conditions of this study not found in other collections."

R3-P2: The absence of a novice/control sample limits any inference about the superiority of the face specialists and whether they are doing anything cognitively different to novices within the context of the study. Ideally, I would prefer it if you collected a novice sample, however that may not be possible. If you cannot collect additional control data this limitation needs to be made clear within the manuscript.

Author response: We acknowledge that this study does not compare specialists to novices, and we now make this explicit in both the Introduction and Discussion. Our judgment was that the behavior of novices was not pertinent to the current study's questions. In the Introduction, we have added: "While such comparisons are valuable, the present study focuses specifically on the cognitive processes of experts rather than the performance gap between specialists and novices" (p. 2). In the Discussion, Efficiency and feature utility subsection, we edited the text to remove ambiguity that direct comparisons to control groups were made in this study, now stating, "While the present study did not include a novice control group, the high efficiency observed among specialists provided converging evidence that expert-level proficiency in facial image comparison is associated with the ability to reach accuracy decisions using limited visual information." (p. 17). We have also added the following limitation to the Discussion: (p. 23): "Additionally, because this study did not include a novice control group, these findings are specific to trained specialists and may not generalize to the general population, who may not possess the same trained sensitivity to diagnostic features such as the ears."

R3-P3: I found the use of the term 'similarity rating' from the benchmarking study very confusing. What you are measuring is how similar the same feature appears between two different people but we typically consider similarity within the context of two images of the same person. In fact, I think you are measuring the typicality or variability of features. I believe using the term similarity will be confusing for readers. I strongly suggest you change the terminology or make it clearer that you are measuring coincidental similarity of features between different people.

Author response: We acknowledge the confusion caused by the term "similarity rating." Accordingly, throughout the manuscript we have replaced this term with "inter-identity similarity rating" to clarify that we are measuring coincidental similarity between different identities. This change was addressed alongside R3-P9 below.

R3-P4: The study focusses solely on how facial identification specialists get things right. I believe it would be useful to reanalyse the data to look at how the specialists also make errors and if there are any useful trends (see below).

Author response: We agree that analyzing errors is a valuable complement to understanding success. The Supporting Information (Fig D) now includes a detailed breakdown of the feature combinations revealed during incorrect decisions. Although the main text focuses on the probability of success — which implicitly provides the error rate as its inverse ($1 - P(\text{success})$)

— we have added a formal reference to these error-specific patterns in the Results section, making the trends associated with unsuccessful identifications transparent. The reference to the error analysis appears in the last paragraph of the Results subsection, "Best features for accuracy" (p. 15). The analysis itself appears in the Supporting Information as Figure D (SI p. 4).

R3-P5: In line 1 you combine facial examiners and facial reviewers as face specialists. This conflation becomes problematic when, in lines 2-3, you state "Research consistently shows that specialists outperform the general population". Firstly, it would be helpful to specify that they outperform the general population in the task of facial image comparison. Secondly, whilst I agree this statement is true for facial examiners, it is not consistently true for facial reviewers, who are a far more diverse group. White et al., in Understanding Professional Expertise in Unfamiliar Face Matching, found that reviewer groups performed worse than controls in over half of the studies analysed. I would suggest lines 2-3 should be changed to clarify that research shows that facial examiners and only some groups of facial reviewers outperform the general population in facial comparison.

Author response: As suggested by the reviewer, the Introduction has been updated to clarify that research shows that facial examiners and only some groups of facial reviewers outperform the general population in facial comparison. This revision appears in the Introduction, paragraph 1 (p. 2).

R3-P6: Lines 27-28, this may be semantics but I do not agree that the issue for examiners is whether they should compare every available facial feature in the images or only prioritise the most information rich. For examiners who are preparing expert forensic reports it is reasonable to expect that they should follow their method and compare all the available features (for reviewers working in high-throughput environments then feature prioritisation during the comparison may be justifiable to improve efficiency, but you are specifically referring to examiners here). The critical issue, in my opinion, is how the examiner or specialist evaluates the significance/diagnosticity/usefulness of the different features they have analysed and compared, in order to reach a conclusion, and how they have decided on the weighting of their observations to derive the conclusion. Unless the goal is to improve speed and efficiency in high throughput contexts (e.g. reviewing 1:N candidate lists) I do not think this argument applies to forensic face examiners. Instead, examiners need to understand how to weight the significance of different features when evaluating findings.

Author response: We appreciate this perspective and agree that thorough evaluation is central to the expert's task. Our study aims to provide empirical data on feature diagnosticity to better understand how specialists weight different features during evaluations. Accordingly, the Introduction and Discussion have been revised to clarify that we are not arguing against comparing all features in a forensic examination. Rather, we describe the behavioral tendency of specialists to prioritize certain features — a finding we present as informing training and efficiency in rapid assessment contexts, not as a recommendation to change procedural standards for forensic reports. The clarification in the Discussion appears in the "Implications for forensic practice" subsection (p. 21).

R3-P7: Line 36: 'we can empirically determine which features are more useful', it is not clear who the 'we' are that you are referring to, is this the general population or do you mean research has empirically determined this? Please clarify.

Author response: Thank you for pointing this lack of clarity out. In this sentence, "we" has been changed to "researchers," as this refers to researchers empirically determining which features are more useful (p. 3).

R3-P8: Line 101-102 it would be helpful to reiterate that the target images are different identities as this is critical to the purpose of collecting the 'similarity ratings'.

Author response: As suggested by the reviewer, we have revised the relevant sentence to make explicit that the target images were always of different people. The sentence "We therefore needed to obtain feature similarity judgments." has been replaced with "The target images were always of different people; therefore, these rating measure coincidental similarity–the extent to which features appear similar across different identities–rather than similarity between images of the same person."

R3-P9: Line 133: 'We tested whether some features tend to appear more similar than others' My understanding is this was only for the different identity target image pairs? If so, it would be helpful to clarify that you are testing whether some features tend to appear more similar between different identities. I think you need to be clear on this throughout the paper as it is a bit of a confusing concept to measure feature similarity only between different identities, because the most similar features for different identities would likely be the least diagnostic for identity (i.e. they are typical or less variable in congruent image conditions). I think it would be helpful elaborate that you are in fact measuring variability or typicality of features between images of different people. I experienced multiple instances throughout the manuscript where I found the concept of the 'similarity ratings' confusing.

Author response: We agree that "similarity rating" was ambiguous and could be misread as measuring similarity between images of the same person. Throughout the manuscript — including section headings, figure captions, and axis labels — we have replaced this term with "inter-identity similarity rating." This terminology change was made throughout the manuscript.

We have also added a statement to the Benchmarking, Procedure section clarifying that these ratings measure coincidental similarity, the extent to which features appear similar across different identities, rather than within the same identity (p. 5).

R3-P10: As well as altering the terminology of the similarity ratings, it would be helpful to elaborate a bit more on the purpose of collecting these similarity ratings and what they are being used to measure (e.g. in the 'Similarity ratings' section starting line 132).

Author response: We agree that a fuller explanation of the similarity ratings was needed. The Benchmarking Results subsection, "Inter-identity similarity ratings" section has been expanded to clarify that these ratings measure coincidental similarity between people and therefore indicate which features are potentially less diagnostic for a given image pair. We

also explain how these ratings were used to construct the predetermined reveal sequences, allowing us to test whether specialists adapt their strategies based on the diagnostic potential of the features in each pair. The expanded explanation appears in the Results, "Inter-identity similarity ratings" subsection, paragraph 1 (p. 7).

R3-P11: Lines 199-206: I found these paragraphs hard to understand. Do you mean that for the different source image pairs you ranked the features for that pair based on similarity then compared the ranking to the mean ranking of similar features for all different source image pairs? It is also not clear from this paragraph what was the purpose of the image pair ranking.

Author response: We thank the reviewer for pointing out the difficulty in understanding. The "Selecting face images" subsection has been clarified to explain that features were ranked within each image pair based on inter-identity similarity, identifying the most and least diagnostic features for that pair. These rankings were then used to construct the predetermined reveal conditions. We have also clarified that the counts of distinct image pairs were reported to demonstrate that the most similar and dissimilar features were distributed broadly across stimuli, rather than concentrated in a few pairs. The clarified paragraph appears in the Methods, "Selecting face images" subsection, paragraph 2 (p. 10).

R3-P12: Lines 221-223: Do you know the level of experience of the face specialists? Can you specify if they were reviewers or examiners? As reviewers and examiners receive different levels of training (Moreton et al. An international survey of applied face-matching training courses) and can perform differently at the group level I think it would helpful to clarify if you can, or highlight that this information is not known.

Author response: We did not collect data on specific roles or years of experience. The "Participants" subsection now states this explicitly (p. 11). We expanded the discussion to acknowledge the differences and as well as the shared elements of their training. The "Limitations and future directions" subsection has been updated to acknowledge differences in training duration and checklist usage between examiners and reviewers, while noting that both groups tend to share the foundational knowledge of facial regions relevant to this study. We appreciate the paper referenced in the reviewer's comment as it provides a thorough breakdown of the types of training received by different specialist groups and is highly appropriate to include in the paper. This expanded discussion of training differences, including the addition of this reference, appears in the Discussion, "Limitations and future directions" subsection (p. 24).

R3-P13: Figure 5: I was surprised to see that accuracy after 1 reveal appears comparable across the three conditions, suggesting that even when shown the most similar feature between the different identity pairs participants were still just as likely to make a correct decision. This suggests that even with just one similar feature the participants were able to extract identity diagnostic information. Could this be due to the task not being difficult enough or a dissociation where similarity ratings are not necessarily equivalent to the diagnosticity of a feature? I think this warrants a mention within the manuscript.

Author response: We agree that accuracy after one reveal was comparable across conditions and that this is not straightforwardly explained by task difficulty or diagnosticity. We removed the language framing the task as inherently more difficult, as we agree that "difficulty" was a design intent rather than a demonstrated result. The comparable accuracy across conditions is qualified by the observation that participants in the "Similar first" condition more frequently elected to reveal a second feature before making an identification decision, meaning that comparable accuracy required more visual information. We revised the text to attribute the choice to reveal additional features to a mechanism of self-regulated accuracy, and describe the higher reveal rate in the "Similar first" condition as a voluntary decision to gather more evidence when the initial feature was less diagnostic, rather than a difference in task difficulty. These revisions appear in the Discussion, "Efficiency and feature utility" subsection (p. 18).

R3-P14: Also, I was surprised in Figure 5 that the CI bounds increase for the probability of a correct response as more features are revealed, and that the average probability dropped after 3 reveals in the self-reveal condition. If the specialists were accruing information from multiple features I would have expected the likelihood of a correct decision to increase and the CI interval to shrink. This suggests to me that the specialists are not accruing and aggregating information from multiple features and may just be relying on the last feature seen, possibly because they are relying on working memory. I wonder if they were to have used a checklist or some other aide memoire they may have got more accurate as more features were revealed. Examiners typically use workbooks or take notes during comparisons which may allow them to accrue information more effectively. Please consider elaborating on this within the manuscript.

Author response: In alignment with this reviewer's observations, the Discussion has been expanded to address the possibility that specialists may not be fully aggregating information across multiple features, relying instead on the most recently revealed feature. We note this is likely an integration issue rather than a working memory failure, as all previously revealed features remained visible on screen. We also discuss how the use of workbooks and checklists in real-world forensic examinations likely mitigates this effect, enabling examiners to more systematically accrue and synthesize evidence. The new paragraph discussing information integration appears in the Discussion, "Efficiency and feature utility" subsection, paragraph 2 (p. 18-19).

R3-P15: Lines 291-293: I think meant to write that noses had "a lower bound that was the second lowest among the features". Also, from the inspection of Figure 6 I think noses actually has the third lowest lower bound (i.e. after chins and necks).

Author response: Thank you for this careful reading. We believe the confusing text due to unnecessary repetition in the original submission: "Noses had one of the lowest probability estimates, with an upper bound that was the second lowest among the features, and had a low upper bound for success." The ending clause — "and had a lower upper bound for success" — is redundant and may have led readers to believe the first clause referred to the lower bound, when it does not. We removed the ending clause; the sentence now reads: "Noses had one of

the lowest probability estimates, with an upper bound that was the second lowest among the features." (p. 14)

This comment also highlighted the need to report the values from Fig. 6 explicitly rather than only graphically. We therefore created a new table in the Supporting Information showing the probability of success and 95% CIs for each feature, referenced in the main text as: "We calculated the probability of providing a correct answer based on which feature was revealed (Fig 6 as well as Table S1 in Supporting Information)." The revised sentence appears in the Results, "Best features for accuracy" subsection (p. 13). The new supplemental table appears in the Supporting Information as Table S1 (SI p. 5).

R3-P16: Lines 297-299: Please elaborate on what you mean here. My interpretation of the high upper bound on other features implies that they can be very diagnostic of identity in some trials but not always, depending on image and subject conditions. I think this roughly aligns with what you have said but it would be good if you could expand on this here or in the discussion.

Author response: Our interpretations align with the reviewer's. To make this explicit, the "Best Features for Accuracy" section now includes an expanded explanation: while some features may not be universally diagnostic, high upper bounds indicate that they can be highly predictive of identity in specific trials, and the width of those intervals reflects substantial variability in feature utility across image pairs. The expanded explanation appears in the Results, "Best features for accuracy" subsection, paragraph 4 (p. 14):

> "This indicates that while these features may not e universally diagnostic, they can be highly predictive of identity in specific trials. The width of some confidence intervals reflects the substantial variability in feature utility across different image pairs."

R3-P17: Lines 300-314: This section focusses on what features were most commonly selected when specialists made correct decisions after one reveal in the self-selecting condition. Due to the low proportion of errors overall there may be insufficient data, but have you looked at what features were picked when specialists made incorrect decisions, and likewise how many reveals were made for incorrect decisions? I think this would be a useful addition either in the body of the results or the SI, as the current results focus solely on how specialists got things right. Errors made by specialists are just as important to understand and help create effective training regimes, presuming there are any useful trends identifiable in the data.

Author response: We agree that understanding feature selection and reveal counts during incorrect decisions is important for developing effective training. The Supporting Information (Fig D) includes a breakdown of feature combinations and reveal counts for incorrect trials, showing the specific features revealed when specialists made errors. The Results section has been updated to formally reference this analysis. We show only up to 2 reveals in this figure because so few trials involved more than 2 reveals. The reference to the error analysis in the main text appears in the Results section, in the final paragraph of the "Best features for

accuracy" subsection (p. 15). The analysis itself appears in the Supporting Information as Figure D (SI p. 4).

R3-P18: Line 370-372: It is implied here that the face specialists in the current study require less visual information than novices. I do not think your data supports this inference as you have not tested novices.

Author response: We agree that our data do not directly compare specialists to novices. Consistent with edits made in response to a similar comment in R3-P3, the referenced passage in the Discussion has been revised to note that our observations are consistent with Dunn et al. (2022) and provide converging evidence that expert proficiency in facial image comparison is associated with high efficiency — while explicitly acknowledging the absence of a novice control group. This revision appears in the Discussion, "Efficiency and feature utility" subsection, paragraph 1 (p. 17).

R3-P19: Lines 379-380: I agree the results support the conclusion that participants were more likely to reveal more than one feature in the similar first condition, which implies some connection between the participant's perceptions of feature usefulness and pre-rated similarity. But the similar first condition does not appear to be anymore inherently difficult than the dissimilar first or self-selection condition, and in fact has the highest probability of an accurate response with only one reveal according to Figure 5. Please amend this paragraph.

Author response: As addressed in conjunction with R3-P13, the "Efficiency and feature utility" subsection has been revised to clarify that while success probabilities remained comparable across conditions, participants in the "Similar first" condition voluntarily revealed more features. This indicates that they required more visual evidence to meet their internal threshold for a decision — a self-regulated rather than an externally imposed difference. The phrase "where the task was inherently more difficult" has been removed, and the revised logic emphasizes that the increased reveal rate reflects a voluntary choice to gather more evidence, not a difference in task difficulty. These revisions appear in the Discussion, "Efficiency and feature utility" subsection (p. 18).

R3-P20: Lines 384-388: My interpretation of your results (particularly from Figure 6) is that some features are more useful than others but this usefulness varies between different trials, and probably varies due to the training/experience/preferences of the specialists (but hard to prove with your data), with the most consistently reliable features being the ear. Being adaptable is therefore very important for specialists, and understanding what is the most diagnostic feature for a given comparison, which may not be the same for another comparison.

To me, this partly answers your question on whether specialists should view isolated features rather than the whole face. In order to select the most useful feature the specialists would need to see the whole face, or at least all the features, at some point rather than just isolated features. I would be interested to hear your thoughts on this.

Author response: The reviewer's interpretation aligns with our own. Because target images remained fully visible throughout the task, specialists could perform a full-face assessment of

the targets first, and the selective reveal process on the probe image then allowed them to verify which specific features were most diagnostic for that particular pair. We have added a clarification to the end of the first paragraph in the Methods, "Experimental design and procedure" subsection reflecting this point (p. 8).

R3-P21: Lines 438-444: I would also suggest that testing on a wider range of stimuli is required, particularly for incongruent image conditions (e.g. a CCTV still to a custody mugshot with mismatched pose, lighting resolution etc.) as these situations are typically encountered by specialists and add extra complexity in assessing the usefulness of facial features.

Author response: We agree this is an important point. The "Limitations and Future Directions" subsection now addresses how image properties limit generalization. We have added the following sentence to the first paragraph of this subsection (p. 23):

> "Testing a wider range of stimuli types will also be needed to form generalized conclusions, especially including stimuli types that are often encountered by specialists such as comparing security-camera footage stills to a mugshot, comparing across poses, lighting conditions, and other factors known to impact matching accuracy in other studies [28-30]."

As part of this addition, we included three new references: Bindemann et al., 2013; Burton et al., 1999; and Jenkins et al., 2011.
